# Integrated Elementomics–Genomics–Metabolomics Analysis Reveals Plasma Biomarker Networks and Diagnostic Potential for Gastric Cancer

**DOI:** 10.3390/metabo16070487

**Published:** 2026-07-10

**Authors:** Ruoyu Li, Guofeng Li, Shilin Chen, Xuejie Lv, Dan Wang, Jianjun Xiang, Yu Jiang, Dong Tan, Chuancheng Wu

**Affiliations:** 1 Department of Preventive Medicine, School of Public Health, Fujian Medical University, Fuzhou 350108, China; 2 The Key Laboratory of Environment and Health, School of Public Health, Fujian Medical University, Fuzhou 350108, China; 3 Fuian Key Laboratory of Environmental Factors and Cancer, School of Public Health, Fujian Medical University, Fuzhou 350108, China; 4 Xiamen Center for Disease Control and Prevention, Xiamen 361013, China

**Keywords:** gastric cancer, plasma biomarkers, genome-wide association study, metabolomics

## Abstract

**Background:** Gastric cancer remains a leading cause of cancer-related deaths worldwide. Although significant progress has been made in clinical diagnosis and treatment, the molecular mechanisms underlying gastric cancer have not yet been fully elucidated. To address this, this study employs a multi-omics approach to systematically analyze the molecular characteristics of gastric cancer. **Methods:** This case–control study enrolled 218 GC patients and 218 healthy controls, and adopted a multi-omics strategy combining inductively coupled plasma mass spectrometry (ICP-MS), element-related genome-wide association study (eGWAS), and untargeted metabolomics to explore the element-gene-metabolite regulatory axis in GC. **Results:** A total of nine plasma differential elements associated with gastric cancer were identified, with a combined diagnostic accuracy of 0.918. Specifically, elements such as Fe, Co, and Li showed significant correlations with 63 genes involved in key signaling pathways, including MAPK, SMAD, and Wnt. Genome-wide association studies (GWAS) revealed that gastric cancer-related genes were significantly enriched in cancer-associated pathways and signaling cascades such as Rap1. Metabolomic analysis further demonstrated that 20 elements in the gastric cancer cohort correlated with 94 metabolites, predominantly enriched in pyrimidine and glutathione metabolism pathways. **Conclusions:** These nine plasma differential elements showed high combined diagnostic efficacy and were associated with genes and metabolites enriched in cancer-related signaling, metabolic reprogramming, and DNA damage response pathways. Together, these findings suggest potential multi-level associations among plasma elemental alterations, genetic variation, and metabolic dysregulation in GC, providing candidate circulating biomarkers and mechanistic clues for future investigation.

## 1. Introduction

Gastric cancer (GC) is one of the most common malignant tumors. According to the latest statistics from GLOBOCAN 2022, it ranks fifth in both incidence and mortality among all cancers [1], making it one of the major contributors to the disease burden. The high incidence and mortality rates of stomach cancer are primarily due to the absence of symptoms in the early stages, which often leads to diagnosis at an advanced stage and a poor prognosis. Traditional methods of gastric cancer screening primarily rely on endoscopy and histopathological examination; however, they have low acceptance rates and offer limited treatment options. Consequently, the importance of analyzing biomarkers in biological tissues—particularly in serum—has become increasingly evident as a means of moving beyond traditional diagnostic and treatment models [2], which contributes to improved early diagnosis and disease management.

Gastric cancer is a disease influenced by both genetic and environmental factors and develops in multiple stages [3]. Environmental factors play a significant role in the development and progression of gastric cancer [4]. Elements found in the environment, particularly geochemical elements in soil, water, and food, are closely linked to human health. Essential trace elements may act as structural components or cofactors of various enzymes and functional proteins, thereby participating in enzymatic regulation, redox homeostasis, immune responses, and DNA damage repair [5,6]. In contrast, exposure to toxic metals such as mercury, cadmium, lead, and arsenic may induce oxidative stress, inflammatory responses, DNA damage, and impaired DNA repair, thereby contributing to genomic instability [7]. As an emerging field of research, elementomics is used to study changes in trace elements under different physiological or pathological conditions. By identifying differential elements, it can be used to screen for biomarkers and aid in disease diagnosis [8], offering a new perspective on understanding the development of diseases such as gastric cancer.

Genetic variations also play a key role in both susceptibility to and progression of gastric cancer. The accumulation of genetic and epigenetic alterations in tumor-associated genes drives the onset and progression of the disease [9]. Single-nucleotide polymorphisms (SNPs) are the most common type of genetic variation and are currently widely used in research related to cancer risk, diagnosis, and prognosis. GWAS have become a key method for identifying disease-associated genetic variants, and a large number of disease susceptibility loci have already been identified. However, traditional GWAS directly link genotypes to clinical phenotypes, which presents challenges such as weak genetic effects and the fact that most loci are located in non-coding regions, making it difficult to determine the impact of gene mutations on biological functions and phenotypes [10]. Against this backdrop, intermediate phenotypes such as transcriptomic, metabolomic, and elementomic data have been progressively incorporated into genome-wide association studies, helping to enhance the biological interpretability of the results and elucidate the underlying mechanisms [11].

In addition to genetic mutations, metabolic reprogramming is a key feature of tumor initiation and progression. Metabolomics enables the systematic detection of small-molecule metabolites, reflecting the downstream biochemical changes induced by genetic mutations, environmental exposures, and tumor metabolic dysregulation [12]. Gastric cancer is often associated with abnormalities in multiple pathways, including energy metabolism, nucleotide metabolism, amino acid metabolism, and redox homeostasis; these changes provide a functional basis for elucidating the disease’s biological characteristics. Therefore, by integrating metabolomics analysis, it is possible to identify metabolic alterations associated with upstream elemental dysregulation and genetic variations, thereby effectively complementing studies in elementomics and genomics.

In summary, trace elements, susceptibility genes, and endogenous metabolites may be interconnected across different biological levels during gastric carcinogenesis. As primary exogenous triggers, environmental trace elements disrupt systemic redox homeostasis and DNA damage response, thereby altering the transcription and function of gastric cancer susceptibility gene [13,14]. In turn, aberrant gene activity further remodels intracellular metabolic pathways and disturbs the homeostasis of small-molecule metabolites, ultimately facilitating the initiation and progression of gastric tumors.

The pathogenesis of gastric cancer involves complex interactions across multiple biological levels, and a single omics study is unlikely to elucidate its underlying mechanisms fully [15]. Although previous studies have separately investigated trace-element alterations [14,16], genetic susceptibility loci [17], and metabolic abnormalities [18] in gastric cancer, integrated analyses linking circulating elemental profiles, genetic variants, and metabolites remain limited [19,20]. To this end, this study employs ICP-MS to analyze plasma elemental profiles quantitatively and combines elemental-related genetic analysis with metabolomics to construct an element–gene–metabolite regulatory network. Through this multi-omics research framework, this study aims to identify potential circulating biomarkers and elucidate the molecular characteristics and mechanisms of gastric cancer.

## 2. Study Population, Materials and Methods

### 2.1. Study Population

This study was approved by the Biomedical Research Ethics Committee of Fujian Medical University (Approval No.: 2014-97). All participants provided written informed consent. All experimental procedures were conducted in accordance with the principles of the Declaration of Helsinki.

#### 2.1.1. Inclusion and Exclusion Criteria for Cases and Controls

The cases were drawn from newly diagnosed gastric cancer cases at Xianyou County Hospital in Fujian Province.

Inclusion criteria: New cases were confirmed pathologically based on tissue specimens obtained via surgery or endoscopy; diagnosis dates ranged between April 2021 and December 2025; all participants were residents of Xianyou for at least 10 years.

Exclusion criteria: (1) Presence of known congenital disorders; (2) Patients with metabolic disorders, severe cardiac, pulmonary, hepatic, renal, neurological, or psychiatric conditions; (3) Pregnant or breastfeeding women; (4) Individuals with alcohol or drug addiction, or those who took proton pump inhibitors, hormones, or nonsteroidal anti-inflammatory drugs (NSAIDs) on a long-term basis; (5) Patients with chronic inflammatory diseases; (6) Individuals who presented symptoms of any acute illness within the past two weeks or who experienced significant stress (e.g., psychological trauma, burns); (7) Individuals with blood disorders (leukemia, anemia).

The control group was matched with the cases by gender and age (±3 years).

Inclusion criteria: Healthy individuals who resided in the same township as the cases or in townships with similar gastric cancer mortality rates were selected as controls; all controls had lived in Xianyou for at least 10 years. Exclusion criteria were the same as those for the cases.

A total of 436 participants were ultimately included in the study, comprising 218 gastric cancer patients and 218 healthy controls. The age range of the gastric cancer group was 42–80 years, with a mean age of (64.84 ± 7.73) years. The age range of the healthy control group was 41–81 years, with a mean age of (65.12 ± 7.98) years. Except for educational level, tea consumption, and pickled vegetable intake, the baseline characteristics of the two study groups were comparable (Table 1).

#### 2.1.2. Blood Sample Collection and Processing

After the subjects provided and signed the informed consent form, 5 mL of peripheral blood was collected after an 8 h fast. Plasma was separated by centrifugation at 3000 rpm for 10 min and stored in a −80 °C freezer pending subsequent analysis.

### 2.2. Plasma Element Analysis

#### 2.2.1. Plasma Sample Pretreatment

Plasma samples were removed from the −80 °C freezer, thawed, and fully mixed before being dispensed into clean digestion tubes. A total of 200 μL of plasma was taken from each sample, followed by the addition of 200 μL of electronic-grade nitric acid (HNO_3_) (Shanghai Aladdin Biochemical Technology Co., Ltd., Shanghai, China) and 400 μL of pure hydrogen peroxide (H_2_O_2_) (Shanghai Aladdin Biochemical Technology Co., Ltd., Shanghai, China). The mixture was diluted to a total weight of 6 g with ultrapure water. Blank tubes were prepared using the same procedure as the sample tubes, with the omission of plasma addition.

#### 2.2.2. Microwave Digestion

The pretreated samples were placed in a microwave digestion system (CEM Corporation, Matthews, NC, USA) for digestion. Upon completion of digestion, the solutions were transferred to centrifuge tubes specific for ICP-MS detection (Agilent Technologies, Inc., Santa Clara, CA, USA). The digestion procedure included a temperature rise to 160 °C with a 10 min holding period, followed by heating to 180 °C and maintaining this temperature for 30 min.

#### 2.2.3. ICP-MS Analysis

Elemental analysis was performed using an ICP-MS instrument (Agilent Technologies, Inc. (U.S.)) under optimized experimental conditions. Argon (Xinzhongming Chemical (Fuzhou) Co., Ltd., Fuzhou, China) was used as the carrier gas with a stabilization time of 30 s, and each sample was measured in triplicate. The standard curve was established with a concentration range of 0–100 μg/L for quantitative analysis. Regression equations for the calibration curve were generated by detecting blank solutions and serially diluted standard solutions (U.S. private equity firm) to ensure the accurate quantification of element concentrations.

### 2.3. Peripheral Blood Genotyping

#### 2.3.1. DNA Extraction

Blood samples were mixed with red blood cell lysis buffer (NH_4_Cl 8.29 g, NH_4_HCO_3_ 0.07 g, EDTA 0.37 g, ddH_2_O) at a ratio of 1:6 and incubated overnight at 4 °C. The mixture was then centrifuged at 4000 rpm for 6 min (Changsha Xiangyi Centrifuge Instrument Co., Ltd., Changsha, China), and the white blood cell pellets were retained. The pellets were resuspended in PBS (Fuzhou Keno Biotechnology Co., Ltd., Fuzhou, China), mixed with DNAzol (Beijing Biotek Biotechnology Co., Ltd., Beijing, China), and pipetted repeatedly to achieve thorough homogenization. Absolute ethanol (Fuzhou Keno Biotechnology Co., Ltd.) was added to precipitate DNA, and the solution was centrifuged at 12,000 rpm for 5 min before the supernatant was discarded. The precipitated DNA was washed twice with 75% ethanol (Fuzhou Keno Biotechnology Co., Ltd.) l and centrifuged again. After air-drying, the DNA was dissolved in double-distilled water in a 55 °C water bath for 2–4 h, and the final DNA samples were stored at −80 °C. DNA concentration was measured using a NanoDrop™ ND-1000 UV-Vis spectrophotometer (NanoDrop Technologies, Inc., Wilmington, DE, USA), and the 260/280 absorbance ratio was recorded to evaluate DNA purity.

#### 2.3.2. Quality Control of DNA Samples

All DNA samples were first detected for concentration and integrity. Qualified samples met the following criteria: a 260/280 ratio ranging from 1.7 to 2.1, a concentration higher than 50 ng/μL, a volume greater than 15 μL, no RNA or protein contamination, and no obvious DNA degradation with clear main electrophoresis bands. Electrophoresis results confirmed that all detected DNA samples were intact and free of protein contamination and degradation.

#### 2.3.3. Genotyping Using the Axiom™ Precision Medicine Research Array

The Axiom^®^ 2.0 Precision Medicine Research Chip (Affymetrix, Inc., Santa Clara, CA, USA) was used for sample genotyping. DNA samples were processed through multiple reagent-based procedures on the Axiom 2.0 system, including denaturation, equilibration, and amplification. During denaturation, DNA samples were mixed with the denaturation buffer. After 10 min of room-temperature incubation, neutralization buffer was added, and the mixture was vortexed (Fuzhou Keno Biotechnology Co., Ltd.) thoroughly for neutralization. Subsequently, 230 μL of amplification mixture was added to each sample well. After complete mixing, samples were incubated in a hybridization oven at 37 °C for 23 h.

Following amplification, DNA samples underwent fragmentation and precipitation. Samples were treated with programmed temperature changes in a preheated hybridization oven (Shanghai Boxun Industrial Co., Ltd., Shanghai, China, Medical Equipment Factory) and fragmented using a fragmentation buffer. Precipitating solution and isopropanol were added for DNA precipitation, and the mixture was centrifuged. The DNA precipitates were washed, dried, and resuspended in buffer solution. All samples were subjected to electrophoresis quality control, which verified intact, degradation-free DNA with clear bands that met experimental requirements.

Qualified DNA samples were transferred for hybridization. Samples were denatured with denaturing solution, neutralized, and hybridized for 24 h in the GeneTitan instrument (Affymetrix, Inc., Santa Clara, CA, USA). Finally, raw genotyping data were obtained through washing, staining, and scanning procedures. Detailed experimental protocols were provided in the Supplementary Materials. Detailed experimental protocols are provided in Supplementary File S1.

### 2.4. Non-Targeted Plasma Metabolite Analysis

#### 2.4.1. Plasma Metabolite Analysis

Plasma samples were removed from the −80 °C freezer and thawed for 3 h. A total of 150 μL of thawed plasma was pipetted, mixed with 300 μL of acetonitrile (Merck KGaA, Darmstadt, Germany), vortexed (Shanghai Qite Analytical Instruments Co., Ltd.) for 30 s, and incubated in a 4 °C refrigerator for 10 mins. An additional 150 μL of acetonitrile was added, and the mixture was vortexed for 30 s and incubated at 4 °C for 3 h to facilitate protein precipitation. The sample solution was centrifuged at 16,000 rcf (Changsha Xiangyi Centrifuge Instrument Co., Ltd.) for 30 min at 4 °C, and 500 μL of the supernatant was collected and filtered through a 0.22 μm filter into a labeled Eppendorf tube. A volume of 100–150 μL of the processed sample was transferred into a 200-μL liner tube and placed in a 2 mL sample vial for subsequent analysis. Quality control (QC) samples were processed alongside each batch of test samples.

Chromatographic conditions: An Agilent SB-C18 column (2.1 × 50 mm, 1.8 μm) was used. The column temperature was set to 37 °C with a flow rate of 0.25 mL/min, and linear gradient elution was performed with a mobile phase ratio ranging from 98:2 (A:B) to 0:100 (A:B).

Mass spectrometry detection was conducted using a mass spectrometer equipped with a TurbolonSpray (ESI) (SCIEX, Framingham, MA, USA) ion source in both positive and negative ion modes. The ion spray voltage was set at 5500 V for positive ion mode and −4500 V for negative ion mode, with nitrogen (Thermo Fisher, Waltham, MA, USA) serving as the carrier gas. Detailed mass spectrometry parameters were listed in the Supplementary Materials. Detailed mass spectrometric parameters are provided in Supplementary File S1.

#### 2.4.2. Metabolic Phenotype Data Processing and Metabolite Identification

Metabolomic raw data were processed using XCMS software (3.20.0). Raw mass spectrometry data were converted to .mzXML format via Agilent MassHunter software (12.0). Peak identification, alignment, matching, and retention time correction were performed using XCMS Online (https://xcmsonline.scripps.edu/, accessed on 10 July 2024). Metabolite structures were identified and annotated by full-scan secondary mass spectrometry with an Agilent q-TOF mass spectrometer (6546). Metabolite information was confirmed by matching mass-to-charge ratios, retention times, and ion modes against the HMDB (https://hmdb.ca/, accessed on 22 June 2024) and Thermo mzCloud (https://www.mzcloud.org/, accessed on 1 November 2025) databases.

### 2.5. Quality Control Analysis

The ICP-MS instrument performance was verified via standard curve calibration and linearity coefficient evaluation to ensure that the limit of detection (LOD) met experimental requirements. All plasma element concentration data were log-transformed and normalized to reduce systematic errors. Samples with total DNA content below 0.6 μg, diffuse electrophoresis bands, or RNA contamination were excluded. The DQC value was adopted to evaluate genotyping quality, and samples with DQC values lower than 0.82 were eliminated. Genotyping was performed in a blinded manner. Samples with a genotyping success rate below 95%, as well as SNP loci and samples that deviated from Hardy–Weinberg equilibrium, exhibited high deletion rates or abnormal heterozygosity and were excluded from subsequent analysis. QC samples were included in each experimental batch. All metabolomic data were log10-transformed and Pareto-normalized to eliminate batch effects and improve data reliability.

### 2.6. Statistical Analysis of Data

All statistical analyses were performed using SPSS 25.0, R (4.2.2), and GraphPad Prism 8.0 software. The Mann–Whitney U test was used to screen elements with significant intergroup differences. Logistic regression analysis was conducted to evaluate the association between differential plasma elements and gastric cancer risk. Spearman’s correlation analysis was applied to assess the correlations among different plasma elements. Lasso regression was further used to identify key element combinations associated with gastric cancer. Receiver operating characteristic (ROC) curves were plotted to evaluate the diagnostic efficacy of the combined element diagnostic model. In addition, principal component analysis (PCA) was performed using Plink software (2.0). GWAS analysis was conducted via GAPIT software (version 3). Gene Ontology (GO) and Kyoto Encyclopedia of Genes and Genomes (KEGG) pathway enrichment analyses of genes related to differential plasma components were performed using the DAVID database. Multiple linear regression analysis was used to explore the associations between plasma elements and metabolites. MetaboAnalyst 5.0 was utilized for pathway enrichment analysis of correlated metabolites, and a multidimensional regulatory network integrating genes, plasma elements, and metabolites was constructed.

## 3. Results

### 3.1. Screening for Differential Elements in Plasma

**Figure 1:**
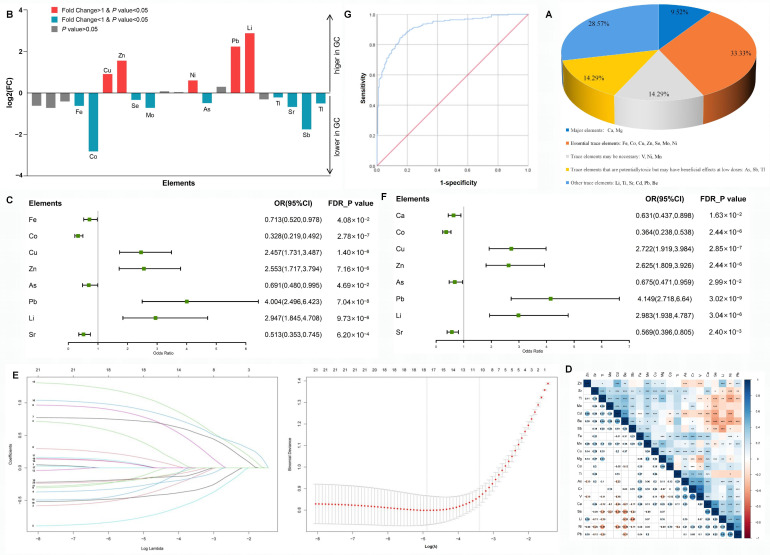
(**A**) Classification map of plasma elements; (**B**) Distribution of different plasma elements between gastric cancer group and control group; (**C**) Forest plot of the results of logistic regression analysis of plasma differential elements; (**D**) Correlation analysis heat map of 21 plasma elements; (**E**) Diagram of Lasso regression results (The trajectory of Lasso regression coefficient change; A 10-fold cross validation graph of the penalty term); (**F**) Forest plot of Logistic regression results based on Lasso regression; (**G**) ROC curve of plasma differential elements for combined diagnosis of GC. In panel (**D**), *, **, and *** indicate *p* < 0.05, *p* < 0.01, and *p* < 0.001, respectively.

Twenty-one elements were quantitatively measured using ICP-MS (Figure 1A). The Mann–Whitney U test was used to analyze and compare differences in plasma element levels between the gastric cancer group and the control group, revealing that the distribution of 14 elements differed between the two groups (Table A1). The levels of Cu, Zn, Ni, Pb, and Li were elevated in the gastric cancer group, while those of Fe, Co, Se, Mo, As, Ti, Sr, Sb, and Tl were reduced in the gastric cancer group (Figure 1B).

### 3.2. The Relationship Between Differential Plasma Elements and the Risk of Gastric Cancer

Using univariate logistic regression and adjusting for confounding factors such as age, sex, and smoking status, we found that 12 plasma biomarkers were associated with the incidence of gastric cancer (FDR_*p* value < 0.05) (Table A2). Further multivariate logistic regression analysis revealed that eight plasma elements—Fe, Co, Cu, Zn, As, Pb, Li, and Sr—were associated with the risk of gastric cancer (FDR_*p* value < 0.05) (Table A3) (Figure 1C).

### 3.3. Identification of Key Plasma Biomarkers for Gastric Cancer

An analysis of the correlations among 21 plasma elements revealed a certain degree of correlation between them (Figure 1D). To address the issue of multicollinearity, Lasso regression was used to identify differentially expressed elements in plasma. The optimal model was ultimately determined through 10-fold cross-validation, resulting in the identification of 10 key elements (Figure 1E). Further logistic regression analysis, adjusted for factors such as age, sex, smoking, and alcohol consumption, revealed that eight plasma elements—Ca, Co, Cu, Zn, As, Pb, Li, and Sr—were most strongly associated with the risk of gastric cancer (Figure 1F).

### 3.4. Evaluation of the Diagnostic Capabilities of Combined Plasma Differential Elements

Using a combination of multivariate logistic regression and Lasso regression, we identified nine plasma elements with significant differences: Ca, Fe, Co, Cu, Zn, As, Pb, Li, and Sr. The ROC curve was used to evaluate the screened plasma differential markers (Figure 1G), with a combined diagnostic accuracy of 0.918, indicating that the nine screened plasma differential markers demonstrate high accuracy and diagnostic value for the combined diagnosis of gastric cancer.

### 3.5. Genome-Wide Association Analysis of Plasma Differential Elements

Using a generalized linear model (GLM), we conducted an association analysis between the 9 plasma differential elements identified in the screening and 265,792 SNP loci, adjusting for age, sex, smoking, alcohol consumption, pickled vegetable intake, and the first three principal components of PCA as covariates. To control the false positive rate, a significance threshold of *p* < 1.88 × 10^−7^ was set using the Bonferroni correction, and a suggestive threshold of *p* < 1.00 × 10^−6^ was established.

The results showed that three plasma elements (Fe, Co, Li) were significantly associated with 41 SNP loci (63 genes) (Table A4). Five plasma elements were associated with 84 SNP loci (121 genes).

**Figure 2:**
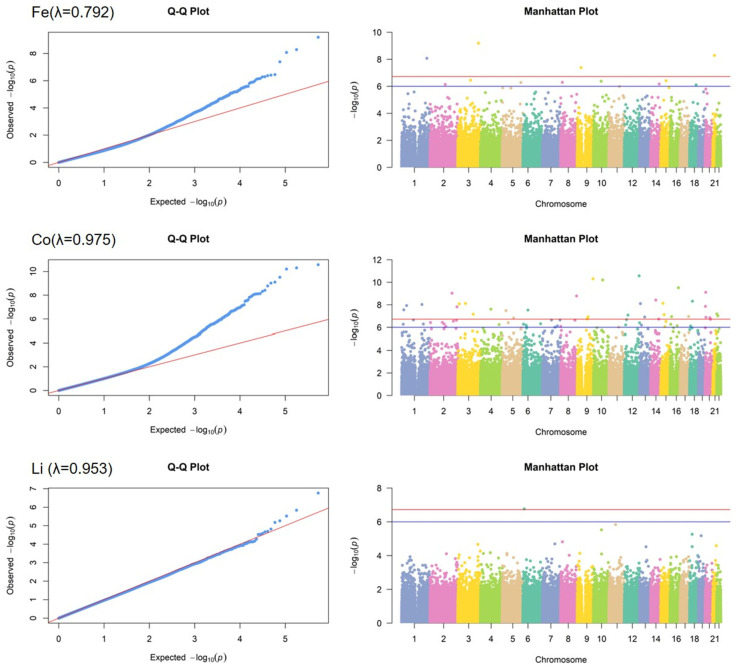
Q-Q plots and Manhattan plots for the genome-wide association analysis of plasma differential elements in gastric cancer. In the Q-Q plots, the *x*-axis represents the expected −log10 (*p* value), and the *y*-axis represents the observed −log10 (*p* value). The blue points represent the observed GWAS p values, and the red diagonal line indicates the expected distribution under the null hypothesis. The genomic inflation factors were Fe (λ = 0.792), Co (λ = 0.975), and Li (λ = 0.953). In the Manhattan plots, each point represents one SNP, and different colors are used to distinguish SNPs located on different chromosomes and do not indicate different levels of statistical significance. The *x*-axis indicates chromosomal position, and the *y*-axis represents −log10 (*p* value). The red and blue horizontal lines indicate the Bonferroni-corrected significance threshold (*p* < 1.88 × 10^−7^) and the suggestive significance threshold (*p* < 1.00 × 10^−6^), respectively.

The results of the GWAS analysis are relatively reliable. The Q-Q plot shows that the observed values align with the expected values, and the inflation factor λ is close to 1, indicating a low probability of false positives. The Manhattan plot illustrates the distribution of genetic mutation sites. The red line represents the threshold for statistical significance (*p* = 1.88 × 10^−7^), while the blue line represents the threshold for suggestive significance (*p* = 1.00 × 10^−6^). Points above these lines indicate SNP loci that are significantly or potentially associated with plasma differential elements in gastric cancer (Figure 2).

### 3.6. Functional Enrichment and Pathway Analysis of Genes Associated with Differentially Expressed Elements in Plasma

**Figure 3:**
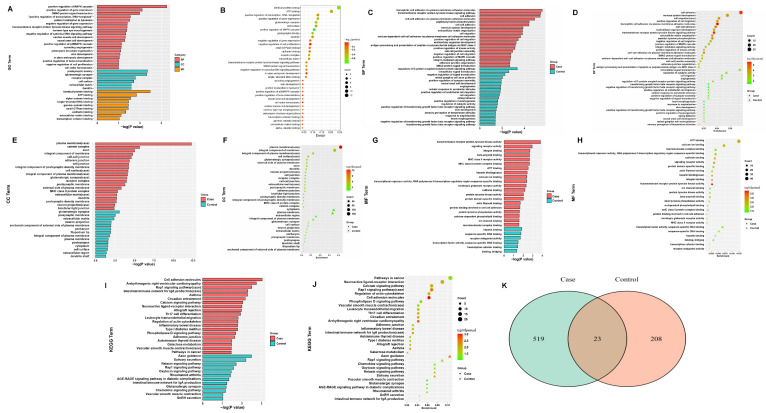
(**A**) Bar chart of GO enrichment results of plasma different-element related genes; (**B**) Bubble plot of enrichment results of differential element-related genes in plasma; (**C**) Bar graph of enrichment of biological functions of genes in two groups; (**D**) Bubble plot of enrichment of biological functions of genes in two groups; (**E**) Bar graph of enrichment of cell component of genes in two groups; (**F**) Bubble plot of enrichment of cell component of genes in two groups; (**G**) Bar graph of enrichment of molecular function of genes in two groups; (**H**) Bubble plot of enrichment of molecular function of genes in two groups; (**I**) Bar graph of enrichment of pathway analysis of genes in two groups; (**J**) Bubble plot of enrichment of pathway analysis of genes in two groups; (**K**) Venn diagrams of two groups of population element-related genes.

We performed a GO enrichment analysis on the 63 associated genes from the GWAS results that met the significance threshold (*p* < 1.88 × 10^−7^) (Figure 3A). These 63 genes are primarily involved in biological processes such as the MAPK cascade, gene transcription regulation, SMAD protein signaling, downregulation of the Wnt signaling pathway, neuronal development, and angiogenesis. These are associated with neuronal dendrites, postsynaptic density, and the extracellular matrix, and their biological functions include protein binding, ATP binding, and DNA binding (Figure 3B).

### 3.7. Genome-Wide Association Analysis of Plasma Elements in Two Populations

A genome-wide association analysis of plasma elements in gastric cancer patients and a control population revealed that, in the gastric cancer group, six plasma elements were significantly associated with 405 SNP loci (542 genes) (Table A5); in the control group, 11 elements were significantly associated with 161 SNP loci (231 genes) (Table A6).

### 3.8. Analysis of the Overlap in Plasma Element-Associated Genes Between the Two Groups

We performed an intersection analysis of the element-associated genes from the two groups. We then defined the set of genes in the gastric cancer group, excluding those shared with the control group, as the gastric cancer group’s element-associated genes, and defined the set of genes in the control group, excluding those shared with the gastric cancer group, as the control group’s element-associated genes. The results showed 519 element-associated genes in the gastric cancer group and 208 in the control group (Figure 3K).

### 3.9. Gene Enrichment and Pathway Analysis for Elements Associated with the Two Groups

Further enrichment and pathway analysis revealed that element-associated genes in the gastric cancer group were primarily involved in biological processes such as cell adhesion, transmembrane receptor tyrosine kinase signaling pathways, neural development, and cell migration, whereas element-associated genes in the control group were primarily involved in processes such as neural development, angiogenesis, and transforming growth factor β receptor signaling pathways. In terms of cellular components, the element-associated genes in the gastric cancer group were primarily associated with axons, dendrites, plasma membranes, and membrane components, synaptic complexes, anchoring structures, and cell junctions, whereas those in the control group were primarily associated with filopodia and glutamatergic synapses. (Figure 3C–F).

In terms of molecular function, element-associated genes in the gastric cancer cohort were primarily associated with transmembrane receptor tyrosine kinase activity, β-amyloid binding, MHC class II receptor activity, and MHC class II protein complex binding; in the control group, element-associated genes were primarily associated with sequence-specific DNA binding and receptor antagonist activity (Figure 3G,H).

In the KEGG pathway enrichment analysis, genes associated with the gastric cancer group were primarily enriched in pathways related to cancer, the Rap1 signaling pathway, the immune network, and circadian rhythm, among others. Genes associated with the control group were primarily enriched in pathways related to axon guidance, the relaxin signaling pathway, and gonadotropin secretion, among others. (Figure 3I,J).

### 3.10. Enrichment Analysis of Gene Pathways Corresponding to Elements in the Two Groups

Compare the gene regulatory patterns associated with each of the 6 elements and 519 genes in the gastric cancer cohort with those of the 11 elements and 208 genes in the control cohort.

**Figure 4:**
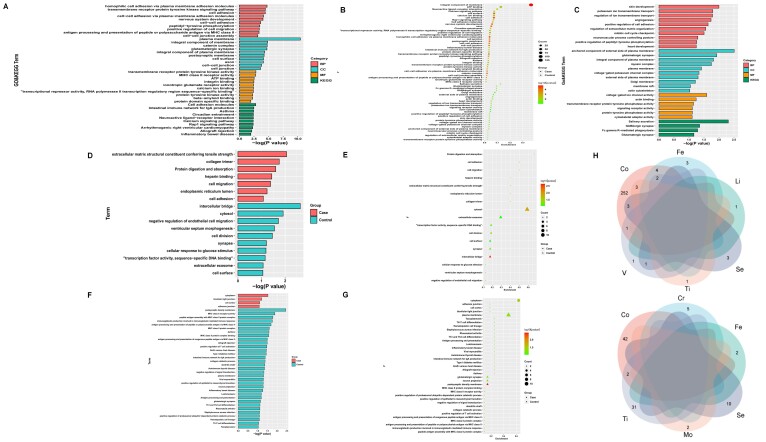
(**A**) Bar graph of Co related gene enrichment in gastric cancer group; (**B**) Bubble plot of Co-related gene enrichment in two groups of population; (**C**) Bar graph of Co related gene enrichment in control group; (**D**) Bar graph of Se-related gene enrichment in two groups of population; (**E**) Bubble plot of Se-related gene enrichment in two groups of population; (**F**) Bar graph of Ti-related gene enrichment in two groups of population; (**G**) Bubble plot of Ti-related gene enrichment in two groups of population; (**H**) Venn diagram of enrichment pathway of element-related genes in two populations (Gastric cancer group and Control group),(Different colors represent gene sets associated with different elements, overlapping regions indicate shared genes, and the numbers indicate the corresponding gene counts).

In the gastric cancer group, genes associated with the Co element were primarily enriched in biological processes such as cell adhesion and transmembrane receptor tyrosine kinase signaling pathways, and were significantly enriched in pathways including the intestinal immune network, circadian rhythm regulation, and calcium signaling pathways. In the control group, genes associated with the Co element were enriched in processes such as angiogenesis, cell adhesion, and the mitotic cycle, and were also enriched in pathways such as GABAergic and glutamatergic synapses (Figure 4A–C).

In the gastric cancer group, genes associated with the Se element were primarily enriched in biological processes such as cell migration and adhesion, as well as in pathways related to protein digestion and absorption; in the control group, they were enriched in processes such as the regulation of endothelial cell migration and cell division, with the molecular function being transcription factor activity (Figure 4D,E).

In the gastric cancer group, the Ti element was associated with cellular components such as tight junctions and the cell cortex, whereas in the control group, it was enriched in pathways related to immune response, antigen processing, and T-cell activation (Figure 4F,G). In the gastric cancer group, Fe-related genes are enriched in the transmembrane receptor tyrosine kinase signaling pathway, primarily associated with ATP binding, and are also enriched in the PI3K-AKT signaling pathway. Pathway analysis of element Li in the gastric cancer group showed enrichment primarily in metabolic pathways, while element V was enriched in cell adhesion regulation and the extracellular matrix.

In the control group, genes associated with chromium were enriched in gene expression regulation and embryonic development, while genes associated with molybdenum were primarily enriched in intracellular signal transduction. Genes associated with beryllium, cadmium, copper, manganese, and lead did not show significant enrichment in any specific pathways.

### 3.11. Analysis of the Intersection of Gene Enrichment Pathways Associated with Population Elements Across the Two Groups

Perform an intersection analysis of the gene enrichment pathways corresponding to each element across the two groups. The results showed that in the gastric cancer group, Co- and Fe-associated genes were jointly enriched in signaling pathways involving transmembrane receptor tyrosine kinases and ATP binding; Co- and Se-associated genes were jointly enriched in cell migration and cell adhesion; Co- and Ti-associated genes were enriched in tight junctions and cell adhesion; and Fe- and V-associated genes were enriched in cellular components such as the plasma membrane. In the control group, only Co- and Ti-related genes were jointly enriched in glutamatergic synapses and plasma membranes, among other cellular components (Figure 4H).

### 3.12. Association Analysis of Plasma Elements and Metabolites

**Figure 5:**
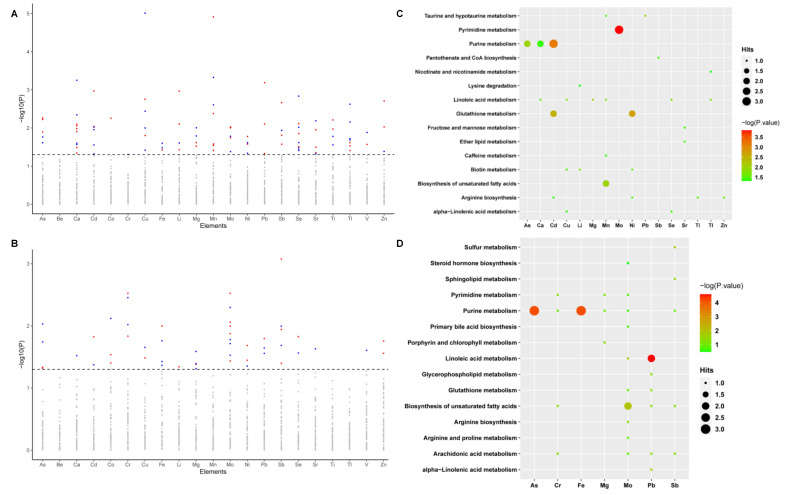
(**A**) Scatter plot of association between plasma elements and metabolites in the gastric cancer population; (**B**) Scatter plot of association between plasma elements and metabolites in the control population; (**C**) Analysis results of plasma element-related metabolite pathways in the gastric cancer population; (**D**) Analysis results of plasma element-related metabolite pathways in the control population. In (**A**,**B**), red and blue points represent significant positive and negative associations, respectively, whereas gray points indicate nonsignificant associations.

This study analyzed 21 plasma elements and, based on structural identification of metabolites, identified 57 plasma metabolites (including 20 nucleotides, 19 lipids, 7 amino acids, 3 peptides, and 8 other metabolites). Multiple linear regression models were constructed to analyze 21 plasma components and 57 metabolites. A *p* value < 0.05 was used as the screening criterion (Table A7 and Table A8) (Figure 5A,B).

### 3.13. Pathway Analysis of Plasma Element-Related Metabolites

The results of the plasma element-metabolite association analysis indicate that, in the gastric cancer cohort, 20 plasma elements were associated with 94 metabolites, while in the control cohort, 17 plasma elements were associated with 51 metabolites. A pathway analysis was performed based on metabolites associated with each plasma element. In the gastric cancer cohort, metabolites associated with Mo were enriched in pyrimidine metabolism; metabolites associated with As, Ca, and Cd were enriched in purine metabolism; metabolites associated with Cd and Ni were enriched in glutathione metabolism; and metabolites associated with Mn were enriched in the biosynthesis of unsaturated fatty acids. In the control group, metabolites associated with Mo were enriched in the biosynthesis of unsaturated fatty acids; metabolites associated with As and Fe were enriched in purine metabolism; and metabolites associated with Pb were enriched in linoleic acid metabolism. These results indicate statistical associations between plasma elements and pathway-annotated metabolites, rather than direct participation of the elements in these metabolic pathways. (Figure 5C,D).

### 3.14. Genome-Wide Association Analysis of Metabolomics Data from Two Cohorts

We conducted a genome-wide association analysis of 57 plasma metabolites against 265,792 SNP loci in gastric cancer patients and control subjects (*p* < 1.00 × 10^−6^). The results showed that, in the gastric cancer cohort, 7 metabolites were associated with 60 SNP loci (82 genes) (Table A9). In the control cohort, 16 metabolites were associated with 67 SNP loci (73 genes) (Table A10).

### 3.15. Analysis of the Intersection of Metabolism-Related Genes Between the Two Groups

**Figure 6:**
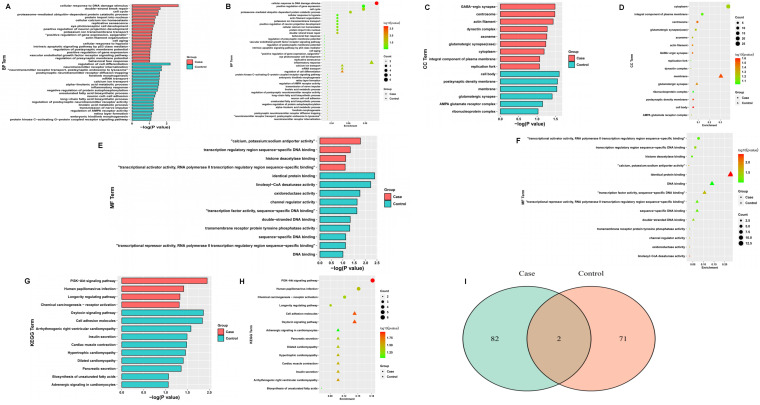
(**A**) Bar graph of biofunctional enrichment of metabolism-related genes in two populations; (**B**) Bubble plot of biofunctional enrichment of metabolism-related genes in two populations; (**C**) Bar graph of cellular components’ enrichment of metabolism-related genes in two populations; (**D**) Bubble plot of cellular components enrichment of metabolism-related genes in two populations; (**E**) Bar graph of molecular function enrichment of metabolism-related genes in two populations; (**F**) Bubble plot of molecular function enrichment of metabolism-related genes in two populations; (**G**) Bar graph of KEGG pathway enrichment of metabolism-related genes in two populations; (**H**) Bubble plot of KEGG pathway enrichment of metabolism-related genes in two populations; (**I**) Venn diagram of metabolism-related genes in two populations.

We performed an intersection analysis of metabolism-related genes between the gastric cancer group and the control group, and then defined the genes not present in both groups as metabolism-related genes specific to the gastric cancer group and the control group, respectively (Figure 6I).

### 3.16. Enrichment Analysis of Metabolism-Related Genes and Pathway Analysis in the Two Groups

Further enrichment and pathway analysis revealed that the metabolism-related genes in the gastric cancer group are primarily involved in biological processes such as the cellular response to DNA damage, the cellular response to hypoxia, the cell cycle, replicative aging, gene expression, and cellular senescence. In the control group, metabolism-related genes were primarily involved in biological processes such as α-linolenic acid metabolism, linoleic acid metabolism, and the biosynthesis of unsaturated fatty acids. In terms of cellular components, the metabolism-related genes in the gastric cancer group are primarily associated with axons and GABAergic synapses. In the control group, metabolism-related genes were primarily associated with postsynaptic dense membranes, AMPA glutamate receptor complexes, and ribonucleoprotein complexes. In terms of molecular function, metabolism-related genes in the gastric cancer group are primarily associated with histone deacetylase binding and transcriptional regulation. In the control group, metabolism-related genes are primarily associated with protein binding, DNA binding, and linoleoyl-CoA lyase activity. KEGG pathway enrichment analysis revealed that metabolism-related genes in the gastric cancer group were primarily enriched in pathways such as the PI3K-AKT signaling pathway, human tumor virus infection, and longevity regulation. In the control group, metabolism-related genes were primarily enriched in pathways such as the biosynthesis of unsaturated fatty acids, arrhythmogenic right ventricular cardiomyopathy, and hypertrophic cardiomyopathy. (Figure 6A–H).

### 3.17. Analysis of the Intersection Between Population Elements and Metabolism-Related Genes

**Figure 7:**
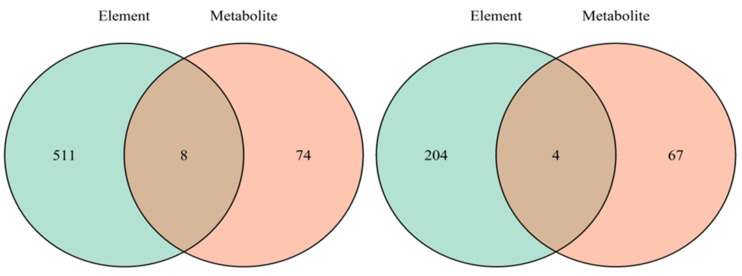
Venn diagram of shared genes of elements and metabolism (Gastric cancer group and Control group).

To explore the associations among genes, elements, and metabolites in the development of gastric cancer, we performed an intersection analysis of element-associated genes and metabolism-associated genes in the gastric cancer group and the control group. The results showed that the gastric cancer group shared 8 elements and metabolic genes, which were associated with 1 element (Co) and 2 metabolites (proline, guanosine diphosphate mannose), which are primarily involved in biological processes such as the DNA damage response. The control group contained 4 genes shared between elements and metabolites, regulating 1 element (Co) and 3 metabolites (proline, methylacetoacetic acid, and DL-dipalmitoylphosphatidylcholine), with their molecular functions primarily enriched in GTPase activity (Figure 7). (Table 2 and Table 3). The two sets of shared genes differ in terms of regulatory elements and metabolites; the shared genes in the gastric cancer group are primarily associated with cobalt and GDP-mannose.

### 3.18. Construction of Gene-Element-Metabolite Association Networks

**Figure 8:**
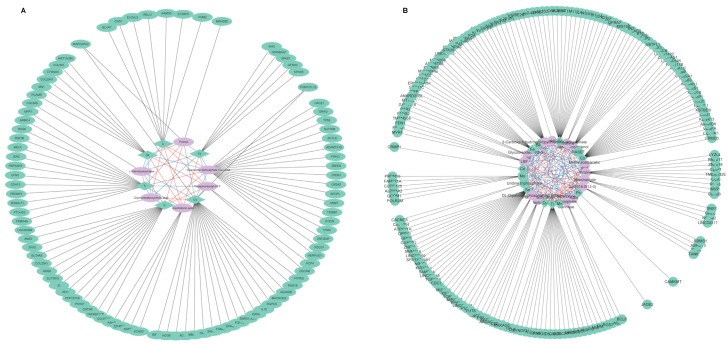
(**A**) plot of gene-element-metabolism regulatory network in gastric cancer; (**B**) plot of gene-element-metabolism regulatory network in control. Green ellipses represent genes, green diamonds represent plasma elements, and purple ellipses represent metabolites. Red and blue edges indicate positive and negative associations, respectively, whereas gray edges indicate gene-related associations.

**Figure 9:**
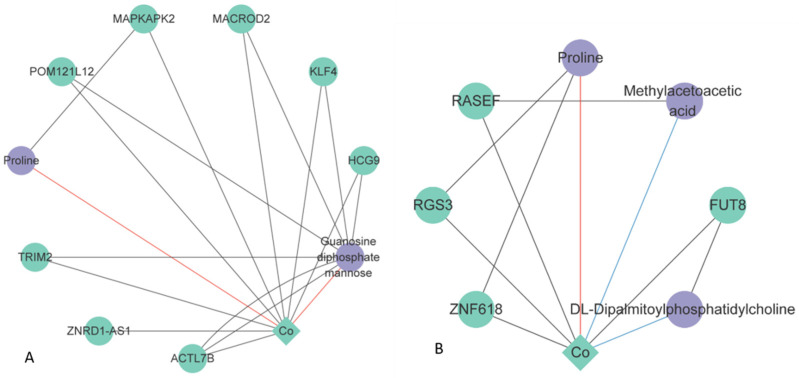
Plot of gene-element-metabolite regulatory network in two populations ((**A**): Gastric cancer group; (**B**): Control group). Teal circular nodes represent genes, teal diamond-shaped nodes represent plasma elements, and purple circular nodes represent metabolites. Red and blue edges indicate positive and negative element–metabolite associations, respectively, whereas gray edges represent gene-related associations.

Based on the above results, we constructed gene-element-metabolite association networks for the gastric cancer cohort and gene-element-metabolite interaction networks for the control cohort (Figure 8A,B), and presented the results for shared gene regulatory elements and metabolites (Figure 9).

## 4. Discussion

Gastric cancer is one of the major global health burdens. Early diagnosis and intervention can effectively reduce incidence rates, improve patient prognosis, and enhance quality of life. Although traditional gastric cancer screening methods, such as endoscopy and histopathological examination, are diagnostically reliable, they are invasive procedures and have low public acceptance. Therefore, the development of new, non-invasive, and well-tolerated screening technologies has become a current research focus. Elemental omics aims to investigate the distribution, concentration, speciation, and biological functions of elements within living organisms. It is currently widely applied in the study of complex diseases and shows great promise for the diagnosis of gastric cancer and the screening of high-risk populations.

Through a combined analysis of plasma elements, genome-wide association studies, and metabolomics, this study identified nine key elements (Ca, Fe, Co, Cu, Zn, As, Pb, Li, Sr). Their combined diagnostic ability was 0.918, indicating high accuracy and demonstrating the potential of elementomics in the early screening of gastric cancer.

### 4.1. The Relationship Between Differential Plasma Elements and the Risk of Gastric Cancer

Calcium is the most abundant mineral in the human body, and calcium homeostasis is closely associated with gastric cancer. Calcium ion (Ca^2+^) levels can influence the cell cycle and energy uptake and play a role in regulating the proliferation, infiltration, invasion, and metastasis of cancer cells [21,22]. Current research on the association between abnormal blood calcium levels and the development of gastric cancer remains controversial. On the one hand, elevated Ca^2+^ levels or increased expression of calcium-binding proteins may promote the development and progression of gastric cancer. Wu et al. pointed out that calcium release activates the PI3K/AKT signaling pathway via ORAI2, which in turn promotes the metastasis of gastric cancer cells through the FAK-MAPK/ERK pathway [23]. On the other hand, it is believed that increased Ca^2+^ levels help suppress tumors. Lin et al. also found that calcium levels were negatively associated with the risk of gastric cancer [16]. This study found that calcium levels in the plasma of gastric cancer patients tend to be lower, suggesting that plasma calcium may be inversely associated with gastric cancer risk; however, the mechanisms underlying this association remain to be further explored.

Iron is an essential trace element for the human body and plays a role in biological processes such as DNA synthesis and repair, heme synthesis, cellular respiration, and immune regulation [24,25,26,27]. The risk of gastric cancer is associated with dietary intake of heme iron [28]. Heme iron can increase the formation of endogenous N-nitroso compounds, which are carcinogenic, and induce lipid peroxidation and DNA damage through reactive oxygen species (ROS) [29], thereby inhibiting apoptosis, thereby inhibiting apoptosis. In addition, ferritin, a marker of iron stores, is inversely associated with the risk of gastric cancer [30], while iron deficiency and anemia may increase oxidative stress and DNA damage, thereby raising the risk of gastric cancer [31]. The results of this study indicate that lower plasma iron levels were associated with an increased risk of gastric cancer and that lower plasma iron levels are associated with an increased risk of gastric cancer.

Cobalt, as an essential component of vitamin B12, promotes iron absorption, supports hematopoiesis, and plays a role in the metabolism of various substances [32]. Studies have shown that cobalt compounds can induce the production of ROS, leading to DNA damage and inhibiting repair [33,34]. In addition, cobalt supports DNA methylation and genetic stability through its interaction with vitamin B12 [35]. Low vitamin B12 levels can impair DNA repair and increase the risk of gastric adenocarcinoma [36]. This study found that plasma cobalt levels were reduced in the gastric cancer group, suggesting that cobalt acts as a protective factor against gastric cancer, consistent with the findings of previous studies.

Copper serves as a cofactor for enzymes essential to basic cellular functions and acts as a key regulator of cellular signaling pathways [37,38], playing a role in biological processes such as cellular respiration, cell proliferation, and angiogenesis [39,40]. Excessively high copper levels can increase the production of toxic free radicals, leading to DNA damage and promoting tumor growth and metastasis [37,41]. The carcinogenic effects of abnormal copper accumulation have been demonstrated in various types of cancer [42,43,44]; for example, copper ions participate in carcinogenic signaling pathways and promote the proliferation and migration of tumor cells [42]. In gastric cancer, high levels of copper are also required to support the proliferation and physiological activity of cancer cells [45]. Liu et al. suggest that low concentrations of copper ions can enhance antitumor activity and inhibit the proliferation of gastric cancer cells [46]. This study shows that elevated copper levels in the gastric cancer group serve as a risk factor for gastric cancer, consistent with the findings of previous studies [16,47].

Zinc is an essential trace element for the human body and plays a role in biological processes such as signal transduction, oxidative stress, immune responses, and DNA damage repair [48,49,50]. Disruptions in zinc homeostasis are closely associated with cancer and cardiovascular disease [51,52]. Several studies have shown that lower blood zinc levels are associated with an increased risk of stomach cancer [53,54,55] al processes such as cellular metabolism, oxidation, and proliferation. Studies have shown that arsenic compounds exhibit significant antitumor activity. For example, arsenic trioxide (ATO) can enhance immunogenic cell death, activate antitumor immune responses [56], and significantly inhibit tumor cell growth [57]. In addition, arsenic sulfide (As_4_S_4_) suppresses the progression of gastric cancer by downregulating the expression of circRNA_ASAP2, thereby inhibiting the activation of the Wnt/β-catenin pathway [58]. Furthermore, this study found that plasma arsenic levels in the gastric cancer group were lower than those in the control group, suggesting that lower arsenic levels may act as a protective factor against gastric cancer, consistent with the findings of other studies [59].

Lead is a toxic metal that poses a health risk due to its carcinogenic properties. Lead can induce the production of ROS [60], increase cellular sensitivity to oxidative stress, and lead to apoptosis [61]. Studies have shown that lead promotes the accumulation of autophagosomes and inhibits lysosomal activity, thereby affecting autophagy and apoptosis [62]. This study found that blood lead levels were significantly higher in the gastric cancer group than in the control group, and that patients with advanced-stage disease had higher blood lead levels than those with early-stage disease, suggesting a potential association between increased blood lead levels and gastric cancer progression.

Lithium is one of the trace elements in the human body, and relevant studies tend to support its anticancer effects. Epidemiological evidence indicates that moderate exposure to lithium in drinking water is significantly associated with a reduced risk of overall cancer and various specific types of cancer [63]. Lithium and its compound LiCl can inhibit pancreatic cancer cell proliferation by suppressing the cAMP pathway and induce apoptosis [64]. In studies on gastric cancer, LiCl has been found to inhibit the Wnt/β-catenin pathway, thereby suppressing the proliferation and invasion of gastric cancer cells [65,66]. This study found that lithium levels were higher in the gastric cancer group than in the control group; no similar findings have been reported in the literature to date, and further investigation into the underlying mechanisms is warranted.

Strontium is also a trace element found in the human body. The results of this study indicate that strontium levels in the gastric cancer group are lower than those in the control group, suggesting that plasma strontium may be inversely associated with gastric cancer risk. Other studies have also shown that strontium levels in the gastric cancer group were lower than those in the control group [67,68]. Strontium may inhibit tumor initiation and progression by protecting chromosomes from damage and reducing DNA damage through the suppression of oxidative stress [69].

### 4.2. Association Analysis of Genes and Plasma Components

To explore potential genetic factors associated with differential plasma elements in gastric cancer, we performed element-quantitative trait locus (eQTL) mapping by correlating nine plasma differential elements with 265,792 SNP loci. The analysis revealed (*p* < 1.88 × 10^−7^) that three plasma elements (Fe, Co, Li) were associated with 41 SNP loci (corresponding to 63 related genes). Two key mutation sites, rs3803357 (associated with susceptibility to acute lymphoblastic leukemia) and rs7199343 (associated with susceptibility to Kawasaki disease), are linked to the *BAHD1* and *ZFHX3* genes, respectively; these genes have been shown to be associated with various cancers.

*BAHD1* is a newly discovered nuclear protein that plays a crucial role in maintaining cell proliferation, differentiation, and the regulation of inflammation. Goryca et al. found that *BAHD1* mutations may be associated with colorectal cancer metastasis [70]. Downregulation of *BAHD1* expression inhibits the proliferation and invasive capacity of breast cancer cells [71]. However, no studies have been found that examine the relationship between *BAHD1* mutations and gastric cancer; our study may provide clues for further research.

*ZFHX3* plays a role in cell proliferation and differentiation and has been identified as a candidate tumor suppressor gene in prostate cancer, non-small cell lung cancer, and other cancers [72,73]; however, it frequently undergoes mutations in cancer, leading to loss of function and an increased tumor mutational burden [74]. Studies have shown that *ZFHX3* promotes breast cancer cell proliferation and tumor growth by regulating the transcription of genes such as *MYC* and *TBX3* [75], but inhibits prostate cancer cell proliferation [76]. This study found that *ZFHX3* is associated with differential elements in gastric cancer plasma; its role in gastric cancer warrants further investigation.

An analysis of enriched pathways for genes associated with plasma differential expression revealed that these primarily involve pathways such as the upregulation of the mitogen-activated protein kinase (MAPK) cascade, SMAD protein signaling, and canonical Wnt signaling. MAPK is a positive regulator of cell proliferation and is upregulated in cancer [77]. Activated MAPK can respond to growth factors in cells, thereby promoting cell proliferation [78], and is associated with the development, metastasis, and prognosis of gastric cancer [79,80]. The SMAD signaling pathway plays a crucial role in the development and progression of cancer, and SMAD protein levels are associated with the suppression of tumor growth and metastasis in various cancers. Halder et al. found that SMAD7 can induce metastasis of colorectal cancer to the liver [75,81]. Other studies have shown that SMAD4 expression is reduced in gastric cancer tissues, while SMAD7 expression is elevated, suggesting that the homeostasis of these proteins in gastric cancer plays a crucial role in tumor cell proliferation, differentiation, apoptosis, and metastasis [82]. The Wnt signaling pathway plays a crucial role in cell proliferation, migration, and apoptosis. When the Wnt signaling pathway is activated, it promotes epithelial–mesenchymal transition via β-catenin, thereby facilitating tumor migration and invasion [83]. Study has shown that when Wnt/β-catenin signaling is inhibited, the cell proliferation cycle is altered. found that when Wnt/β-catenin signaling is inhibited, the cell proliferation cycle is altered, affecting the progression of gastric cancer [84]. Research on these pathways is relatively well-established, but the mechanisms by which these elements influence cancer-related pathways—and thereby contribute to the development of gastric cancer—by modulating their levels or valence states still require further investigation.

In the gastric cancer group, element-associated genes were primarily enriched in pathways related to tyrosine phosphorylation, β-amyloid binding, and cancer, whereas in the control group, they were enriched in pathways related to cell migration and neurodevelopment.

Tyrosine phosphorylation plays a role in regulating cellular signal transduction and key cellular functions [85]. Tyrosine kinases are key molecules in signaling cascades; by inhibiting cell proliferation and promoting apoptosis, abnormalities in the regulation of tyrosine kinases have been identified in the onset and progression of cancer [86,87]. The phosphorylation of peptidyl-tyrosine can be enriched through interactions with metal ions; Fe^3+^ and Ti^4+^ exhibit a preference for phosphate groups [88]. This study found that genes associated with Fe, Ti, and Co were enriched in the biological process of peptidyl-tyrosine phosphorylation, suggesting a potential link between these plasma elements and signaling pathways related to cell proliferation and metastasis in gastric cancer. The specific mechanisms require further experimental validation.

We performed pathway enrichment analysis on the genes associated with each element and conducted an intersection analysis of the pathway results. The results showed that, in the gastric cancer group, Co, Fe, and Se were primarily enriched in biological processes such as the regulation of the transmembrane receptor tyrosine kinase (RTP) signaling pathway and heparin binding.

RTP is a transmembrane protein that mediates signal transduction through the phosphorylation of cellular substrates, regulating cell proliferation, differentiation, apoptosis, and migration; alterations in RTP have been observed in various cancers [89]. In gastric cancer research, the human hepatocellular carcinoma receptor (Eph) is a subfamily of transmembrane protein-tyrosine kinase receptors that can influence immune system function. Studies have shown that Eph overexpression is associated with the aggressiveness of gastric cancer and poor prognosis, and promotes metastasis and invasion in gastric cancer [90]. As mentioned earlier, genes associated with Co, Fe, and Ti are primarily enriched in biological processes involving the phosphorylation of tyrosine residues, while genes associated with Co and Fe are primarily enriched in biological processes regulated by the transmembrane RTP signaling pathway. These findings suggest that genes associated with Co, Fe, and Ti may be linked to tyrosine kinase signaling and peptidyl-tyrosine phosphorylation in gastric cancer. However, whether it promotes the tumorigenesis and metastasis of gastric cancer and the specific underlying mechanisms remain to be further validated.

### 4.3. The Relationship Between Metabolomics and Plasma Elements

The study found that 20 plasma elements were associated with 94 metabolites in the gastric cancer group, while 17 plasma elements were associated with 51 metabolites in the control group. Metabolic pathway analysis revealed that the primary metabolic pathways in the gastric cancer group were the pyrimidine and glutathione metabolic pathways. Pyrimidine metabolism is a component of nucleotide metabolism and is essential for DNA and RNA synthesis. The ability of malignant tumor cells to reprogram metabolic pathways is a key characteristic of cancer, and dysregulation of pyrimidine metabolism is closely associated with cancer progression [91]. Cancer cells maintain deoxyribonucleotide triphosphate (dNTP) levels by reprogramming metabolic pathways, thereby supporting their unlimited proliferation. In addition, mutations in the key oncogenes *TP53* and *MYC* can interact with pyrimidine metabolism genes, upregulating their expression to maintain elevated levels of dNTPs and ensure that the raw materials and energy required for tumor growth are met [92,93].

Cancer cells use metabolic reprogramming to obtain the nutrients and energy needed to support their proliferation, while reducing oxidative stress-induced cellular damage [93]. Glutathione plays a crucial role in antioxidant stress, helping cancer cells cope with higher levels of reactive oxygen species (ROS) to support their increased metabolic and proliferative capabilities [94]. At the same time, their antioxidant capacity also needs to be further enhanced; numerous studies have shown that alterations in glutathione metabolism are associated with programmed cell death in cancer cells [95]. This study found that Mo, Cd, and Ni were associated with metabolites enriched in pyrimidine and glutathione metabolism. These associations may reflect alterations in nucleotide synthesis and redox-related metabolic processes in gastric cancer. However, whether these elements directly affect these pathways or contribute to tumor progression requires further mechanistic validation.

Enrichment analysis of metabolism-related genes in the gastric cancer group and the control group revealed that metabolism-related genes in the gastric cancer group were primarily enriched in pathways related to the cellular response to hypoxia, the PI3K-AKT signaling pathway, the vascular endothelial growth factor receptor signaling pathway, and GABAergic synapses.

Hypoxia is one of the hallmarks of cancer; it promotes tumor metastasis, increases resistance to chemotherapy, and is associated with poor prognosis in cancer patients. Hypoxia-inducible factor 1 (HIF-1) is the primary effector of hypoxia and plays a role in cancer cell proliferation, differentiation, migration, angiogenesis, and increased drug resistance [96]. Research has shown that the expression of the HIF-1 gene in gastric cancer can influence HIF-1 levels, thereby contributing to the process of hypoxia in cancer, and may serve as an effective therapeutic target for cancer [97]. Angiogenesis is a fundamental process in malignant tumors, and vascular endothelial growth factor (VEGF) and the VEGF receptor pathway are key regulators of this process [98]. When cancer cells experience oxidative stress, HIF-1 transcription is activated, promoting VEGF expression and its binding to VEGF receptors on the surface of endothelial cells, thereby facilitating tumor angiogenesis and regulating the proliferation, differentiation, and migration of vascular endothelial cells [99].

The PI3K/AKT signaling pathway is a major downstream signaling pathway of many growth factor receptors and one of the most active signaling pathways in human tumor; it promotes tumor cell proliferation, apoptosis, and invasion through the phosphorylation of PI3K and AKT proteins [100]. Activation of the PI3K/AKT pathway promotes glycolysis, enhances cancer cell survival and proliferation, and inhibits apoptosis and autophagy [101]. During cancer cell metastasis, the PI3K/AKT pathway promotes epithelial–mesenchymal transition (EMT) and stimulates angiogenesis, thereby creating an environment conducive to tumor migration [102].

GABAergic synapses, as a major neural pathway for the transmission of inhibitory neurotransmitters, were initially associated with neurological disorders such as gliomas. Research suggests that these neurotransmitters may play a key role in tumor growth and that tumor cells may regulate their growth through neural innervation [103]. B cells can release GABA, promoting the differentiation of monocytes into anti-inflammatory macrophages, and suppressing antitumor responses by secreting interleukin-10 (IL-10), indicating that neurotransmitters and the immune system interact in tumor initiation and progression [104]. The study also found that GABA accumulates abnormally in lung and colon cancers, but there is limited research on this in gastric cancer. This study found that genes associated with gastric cancer metabolism are linked to GABAergic synapses, suggesting a potential link between metabolism-related genes, neurotransmitter-related pathways, immune regulation, and gastric cancer progression.

### 4.4. Enrichment Analysis of Genes Shared by Elements and Metabolites

An intersection analysis of element- and metabolism-related genes between the gastric cancer group and the control group revealed that the gastric cancer group contained 8 shared element- and metabolism-related genes, which were associated with 1 element (Co) and 2 metabolites (proline and mannitol-1,6-diphosphate), and are primarily involved in biological processes related to the cellular response to DNA damage.

One of the mechanisms by which cellular senescence inhibits tumorigenesis [105]. The DNA damage response (DDR) is a cellular signaling cascade that detects genotoxic stimuli and generates a response to maintain genomic stability and cellular homeostasis [106,107]. p53 is a key tumor suppressor gene; it is activated by DNA damage and hypoxia, promoting cellular senescence and apoptosis, maintaining cellular and genomic stability, and suppressing the development of cancer [108]. We hypothesize that the eight shared genes associated with Co and guanosine diphosphate mannose may be linked to DNA damage response-related processes in gastric cancer. However, the direction and functional significance of these associations require further experimental validation. These findings provide a preliminary mechanistic clue linking plasma elements, metabolites, and DNA damage response-related processes in gastric cancer.

Since this study was based on circulating biomarkers, it is important to consider whether these plasma alterations may reflect tissue-level abnormalities. Although the present study was based on plasma samples, the dysregulated elements, gene-associated signals, and metabolites identified in blood may partly reflect tissue-level alterations in gastric cancer. Previous tissue-based studies have shown that trace element imbalance, cancer-related signaling pathways such as MAPK, Wnt, PI3K/AKT, and VEGF, as well as metabolic reprogramming involving nucleotide metabolism and redox metabolism, are also present in gastric cancer tissue [109,110]. Therefore, the plasma element-gene-metabolite network identified in this study may provide a non-invasive indication of molecular and metabolic abnormalities in gastric cancer tissues. However, the direct concordance between plasma and tissue alterations still requires further validation.

From a clinical perspective, the integrated plasma element-gene-metabolite findings may have potential implications for non-invasive gastric cancer screening. This study established a novel plasma element-gene-metabolite association network for gastric cancer and screened nine differential plasma elements with excellent diagnostic efficiency (AUC = 0.918). Clinically, this plasma elemental panel may serve as a non-invasive auxiliary tool for gastric cancer screening and risk stratification, especially in high-risk populations or individuals with poor acceptance of endoscopy. As the detection of plasma elements is relatively convenient and minimally invasive, it may help identify individuals who require further endoscopic examination, thereby improving the efficiency of early gastric cancer screening. However, before clinical application, further studies are still needed. First, large-scale, multicenter prospective cohorts should be conducted to externally validate the diagnostic performance and determine clinically applicable cutoff values. Second, paired plasma and gastric tissue samples should be analyzed to confirm the consistency between circulating biomarkers and tissue-level dysregulation. Third, in vitro and in vivo experiments are required to clarify the molecular mechanisms linking key elements to gene expression, metabolic reprogramming, and gastric carcinogenesis.

Nevertheless, several factors should be considered before translating these findings into clinical practice. Plasma element levels may be affected by dietary habits, food composition, environmental exposure, and potential pollutant sources. Therefore, the elemental profiles identified in this study may partly reflect the characteristics of this specific population and require further validation in populations from different geographical and cultural backgrounds. In addition, cancer stage may also influence plasma element levels; thus, future studies should perform stage-stratified analyses to further evaluate the clinical applicability of this elemental panel.

Although the identified plasma elemental panel showed good diagnostic performance in this study, its clinical utility for identifying high-risk individuals remains to be further validated. The observed elemental alterations may not be specific to gastric cancer and could also occur in other malignancies or disease conditions, which may affect its diagnostic specificity in clinical practice. Therefore, future studies should include patients with other cancers and benign gastric diseases to evaluate the specificity and differential diagnostic value of this elemental panel. In addition, the measurement of trace elements usually requires specialized analytical instruments, which may limit its immediate application in routine clinical laboratories. Further standardization of detection methods and validation in clinical settings are needed before clinical implementation.

The biological interpretation of these findings should be made with caution. It should be noted that the associations observed in this study represent statistical correlations rather than direct causal relationships. The element-gene and element-metabolite networks indicate that specific plasma elements were associated with genes, metabolites, or enriched biological pathways, but they do not prove that these elements directly regulate these pathways. These findings may reflect broader metabolic disturbances, oxidative stress, inflammatory responses, or indirect biological effects. Therefore, further mechanistic studies are required to clarify whether and how these elements influence specific biological pathways in gastric cancer.

## 5. Conclusions

In this study, 9 plasma elements, including Ca, Fe, Co, Cu, Zn, As, Pb, Li, and Sr, showed high combined diagnostic performance for distinguishing gastric cancer patients from healthy controls, suggesting their potential value as candidate circulating biomarkers for gastric cancer. Multi-omics integration further revealed that element-associated genes and metabolites were enriched in biological processes related to cancer-associated signaling, metabolic reprogramming, and DNA damage response. These findings provide preliminary clues for understanding the potential links among plasma elemental alterations, genetic variation, and metabolic dysregulation in gastric cancer. However, the associations identified in this study do not establish direct causal relationships, and further multicenter validation, paired plasma-tissue analysis, and functional experiments are needed to confirm their clinical utility and underlying biological mechanisms.

## Figures and Tables

**Table 1:** Basic characteristics of the research object.

	Control (*n* = 218)	Case (*n* = 218)	χ2	*p* Value
Age			0.749	0.387
≤65 years old	114	123		
>65 years old	104	95		
Gender			0.109	0.742
Female	54	57		
Male	164	161		
Educational Level			8.892	0.012 *
Elementary school or below	149	176		
Junior high school	43	25		
High school or above	26	17		
Smoking			0.037	0.848
No	100	102		
Yes	118	116		
Alcohol consumption			2.188	0.139
No	180	191		
Yes	38	27		
Tea consumption			25.987	<0.001
No	158	199		
Yes	60	19		
Pickled vegetable intake			17.590	<0.001
No	61	26		
Yes	157	192		
*Helicobacter pylori* infection			0.763	0.382
No	131	122		
Yes	87	96		
Marital Status			0.200	0.655
Married	191	194		
Other	27	24		
Occupation Category			0.455	0.500
Farmer	169	163		
Other	49	55		

Note: * *p* < 0.05 indicates a statistically significant difference between the case group and the control group.

**Table 2:** Shared genetic Information of elements and metabolites in gastric cancer group.

Shared Gene	Elements	*p* Value	Metabolites	*p* Value
*MAPKAPK2*	Co	8.09 × 10^−14^	Proline	3.10 × 10^−7^
*TRIM2*	Co	9.34 × 10^−11^	Guanosine diphosphate mannose	1.36 × 10^−8^
*HCG9*	Co	4.31 × 10^−12^	Guanosine diphosphate mannose	9.31 × 10^−7^
*ZNRD1-AS1*	Co	4.31 × 10^−12^	Guanosine diphosphate mannose	9.31 × 10^−7^
*POM121L12*	Co	7.22 × 10^−11^	Guanosine diphosphate mannose	1.38 × 10^−8^
*KLF4*	Co	2.00 × 10^−11^	Guanosine diphosphate mannose	1.14 × 10^−8^
*ACTL7B*	Co	2.00 × 10^−11^	Guanosine diphosphate mannose	1.14 × 10^−8^
*MACROD2*	Co	1.10 × 10^−13^	Guanosine diphosphate mannose	1.82 × 10^−7^

**Table 3:** Shared genetic Information of elements and metabolites in control group.

Shared Gene	Elements	*p* Value	Metabolites	*p* Value
*RASEF*	Co	2.84 × 10^−10^	Methylacetoacetic acid	9.21 × 10^−7^
*ZNF618*	Co	2.06 × 10^−7^	Proline	6.19 × 10^−7^
*RGS3*	Co	2.06 × 10^−7^	Proline	6.19 × 10^−7^
*FUT8*	Co	6.81 × 10^−8^	DL-Dipalmitoylphosphatidylcholine	8.70 × 10^−7^

## Data Availability

The data presented in this study are available on request from the corresponding author. The data are not publicly available due to restrictions privacy.

## Supplementary Materials

The following supporting information can be downloaded at: https://www.mdpi.com/article/10.3390/metabo16070487/s1, Supplementary File S1: Detailed experimental procedures for peripheral blood genotyping and non-targeted plasma metabolite analysis.

## Appendix A

**Table A1:** Basic information on differential plasma elements (μg/L).

Metal Conc.	Overall (*n* = 436)	Control (*n* = 218)	Case (*n* = 218)	*p*	*p*_Value ^a^
Fe	2410.00 (1521.17, 3045.91)	2709.35 (1853.38, 3504.67)	2040.92 (1357.91, 3024.60)	<0.001	<0.001
Co	0.77 (0.60, 1.11)	0.81 (0.60, 0.98)	0.76 (0.59, 0.98)	0.001	0.001
Cu	1271.49 (1042.49, 1440.89)	1212.34 (1016.83, 1377.34)	1311.25 (1110.38, 1538.09)	<0.001	<0.001
Zn	2159.78 (1241.76, 3772.42)	1633.71 (1003.58, 3112.74)	2912.59 (1630.69, 5225.96)	<0.001	<0.001
Se	121.66 (90.29, 143.00)	128.13 (88.17, 156.82)	119.67 (90.40, 135.45)	0.028	0.028
Mo	3.81 (1.95, 7.19)	4.52 (2.24, 9.73)	3.19 (1.84, 7.15)	0.001	0.001
Ni	1.56 (0.79, 10.88)	1.02 (0.76, 10.73)	8.28 (0.85, 14.41)	<0.001	<0.001
As	33.46 (27.66, 41.13)	35.45 (28.80, 42.64)	32.12 (26.56, 40.31)	0.005	0.006
Pb	4.30 (0.93, 21.05)	2.13 (0.78, 5.55)	8.68 (1.47, 20.02)	<0.001	<0.001
Li	3.22 (1.28, 7.62)	1.74 (1.07, 4.69)	6.97 (2.01, 18.89)	<0.001	<0.001
Ti	678.07 (300.21, 894.08)	684.07 (401.98, 968.22)	645.86 (188.84, 882.91)	0.015	0.015
Sr	76.44 (60.62, 91.11)	80.43 (66.78, 98.56)	69.29 (55.70, 89.46)	<0.001	<0.001
Sb	0.46 (0.14, 1.90)	1.10 (0.12, 9.28)	0.36 (0.14, 1.42)	<0.001	<0.001
Tl	0.43 (0.22, 1.34)	0.53 (0.28, 1.85)	0.33 (0.19, 1.34)	<0.001	<0.001

^a^: *p*-values for significance after log-transformation and standardization of element concentrations.

**Table A2:** Univariate Logistic regression model of plasma differential elements and risk of gastric cancer.

Elements	Unadjusted Model ^a^		Adjusted Model ^b^	
	OR (95%CI)	FDR_*p* Value	OR (95%CI)	FDR_*p* Value
Fe	0.761 (0.617, 0.937)	1.11 × 10^−2^	0.774 (0.627, 0.955)	1.84 × 10^−2^
Co	0.541 (0.427, 0.686)	9.50 × 10^−7^	0.521 (0.405, 0.671)	1.32 × 10^−6^
Cu	1.349 (1.109, 1.640)	3.29 × 10^−3^	1.407 (1.147, 1.726)	1.40 × 10^−3^
Zn	2.433 (1.939, 3.054)	6.68 × 10^−14^	2.486 (1.968, 3.140)	8.76 × 10^−14^
Se	1.639 (1.205, 2.229)	2.19 × 10^−3^	1.666 (1.208, 2.299)	2.24 × 10^−3^
Ni	1.412 (1.166, 1.710)	6.14 × 10^−4^	1.446 (1.186, 1.764)	4.65 × 10^−4^
As	0.800 (0.660, 0.967)	2.29 × 10^−2^	0.805 (0.660, 0.981)	3.15 × 10^−2^
Pb	2.829 (2.216, 3.612)	4.25 × 10^−16^	2.990 (2.304, 3.879)	1.01 × 10^−15^
Li	3.250 (2.509, 4.209)	5.15 × 10^−18^	3.489 (2.629, 4.630)	6.04 × 10^−17^
Sr	0.586 (0.477, 0.719)	9.50 × 10^−7^	0.589 (0.477, 0.728)	2.36 × 10^−6^
Sb	0.645 (0.530, 0.786)	2.72 × 10^−5^	0.661 (0.539, 0.811)	1.45 × 10^−4^
Tl	0.692 (0.567, 0.843)	4.47 × 10^−4^	0.692 (0.564, 0.8419)	6.08 × 10^−4^

^a^: Unadjusted model; ^b^: Adjusted for age, sex, and smoking status.

**Table A3:** Multivariate Logistic regression model of plasma differential elements and risk of gastric cancer.

Elements	Odds Ratio	95%CI	FDR_*p* Value
Fe	0.713	(0.520, 0.978)	4.08 × 10^−2^
Co	0.328	(0.219, 0.492)	2.78 × 10^−7^
Cu	2.457	(1.731, 3.487)	1.30 × 10^−6^
Zn	2.553	(1.717, 3.794)	7.16 × 10^−6^
As	0.691	(0.480, 0.995)	4.69 × 10^−2^
Pb	4.004	(2.496, 6.423)	7.04× 10^−8^
Li	2.947	(1.845, 4.708)	9.73 × 10^−6^
Sr	0.513	(0.353, 0.745)	6.20 × 10^−4^

**Table A4:** Results of genome-wide association analysis of plasma differential elements.

Elements	dbSNP	Chromosome	Alt	Ref	*p* Value	Associated Gene
Fe	rs2111397	3	C	T	6.42 × 10^−10^	*PEX5L*
	rs7275831	21	C	T	5.18 × 10^−9^	*ADAMTS5*, *MIR5009*
	rs7512221	1	T	C	8.42 × 10^−9^	*RAB3GAP2*, *MARK1*
	rs16918479	9	A	G	4.09 × 10^−8^	*TAF1L*, *TMEM215*
Co	rs117426959	12	A	G	2.67 × 10^−11^	*GLT1D1*
	rs35443779	9	G	A	4.87 × 10^−11^	*RXRA*
	rs75430598	10	T	C	6.19 × 10^−11^	*ZMIZ1-AS1*, *LINC00595*
	rs7199343	16	T	A	3.05 × 10^−10^	*ZFHX3*
	rs4815697	20	G	A	7.77 × 10^−10^	*ADRA1D*, *PRNP*
	rs13411587	2	C	T	9.40 × 10^−10^	*MYO1B*, *NABP1*
	rs34763544	8	-	C	1.65 × 10^−9^	*COL22A1*
	rs3783728	14	G	A	3.81 × 10^−9^	*MTHFD1*, *MIR548AZ*
	rs141445749	18	-	TG	4.78 × 10^−9^	*CDH2*, *CHST9*
	rs3803357	15	A	C	7.28 × 10^−9^	*BAHD1*
	rs77879696	3	A	G	7.62 × 10^−9^	*KBTBD8*, *LRIG1*
	rs9512056	13	T	C	7.84 × 10^−9^	*SHISA2*, *ATP8A2*
	rs307594	3	C	T	8.18 × 10^−9^	*SYN2*
	rs143137135	1	A	G	9.28 × 10^−9^	*SEC16B*
	rs6954	1	C	T	1.17 × 10^−8^	*SZT2*
	rs1578568	20	G	A	1.38 × 10^−8^	*PDYN*, *STK35*
	rs4663152	2	T	C	1.52 × 10^−8^	*ARL4C*, *SH3BP4*
	rs75585297	4	A	G	2.44 × 10^−8^	*CCSER1*
	rs3811399	1	A	G	2.80 × 10^−8^	*IFFO2*
	rs3025032	6	C	T	2.94 × 10^−8^	*VEGFA*
	rs4867446	5	T	G	3.27 × 10^−8^	*ZFR*, *SUB1*
	rs5994192	22	G	A	6.42 × 10^−8^	*CECR1*
	rs12636613	3	G	A	6.96 × 10^−8^	*EPHB1*
	rs149719655	15	C	-	7.52 × 10^−8^	*GABPB1-AS1*, *USP8*, *MIR4712*
	rs11051764	12	T	C	8.15 × 10^−8^	*BICD1*, *RNU6-78P*
	rs2072006	22	G	A	9.68 × 10^−8^	*MYO18B*
	rs142547549	17	T	C	1.09 × 10^−7^	*DNAI2*
	rs7206058	16	G	T	1.12 × 10^−7^	*SHISA9*, *ERCC4*
	rs72742855	9	C	T	1.18 × 10^−7^	*ROR2*, *NFIL3*, *MIR3910-1*
	rs11431784	13	A	-	1.22 × 10^−7^	*LINC00448*
	rs1884897	20	G	A	1.30 × 10^−7^	*CASC20*, *BMP2*
	rs74810718	20	G	A	1.42 × 10^−7^	*CHD6*, *PTPRT*
	rs58845989	5	T	C	1.49 × 10^−7^	*LOC100133050*
	rs76944556	9	G	A	1.78 × 10^−7^	*FRMD3*, *RASEF*
	rs3091524	20	G	A	1.79 × 10^−7^	*SLC2A10*, *EYA2*
	rs10690505	14	TTA	-	1.85 × 10^−7^	*KCNK13*
Li	rs976081	6	T	G	1.72 × 10^−7^	*ELOVL2*

Note: dbSNP, Database of Single Nucleotide Polymorphisms; Alt, alternative allele; Ref, reference allele.

**Table A5:** Genome-wide association analysis of 21 plasma elements in gastric cancer.

Elements	dbSNP	Chromosome	Alt	Ref	*p* Value	Associated Gene
Co	rs10095740	8	T	C	1.05 × 10^−17^	*DLGAP2*
	rs141445749	18	-	TG	2.22 × 10^−17^	*CHST9*, *CDH2*
	rs78335066	4	C	T	8.41 × 10^−16^	*PCDH7*
	rs12740417	1	T	C	2.49 × 10^−15^	*NCF2*
	rs77658897	1	G	A	5.67 × 10^−15^	*RGS18*
	rs117415976	4	A	G	5.90 × 10^−15^	*PHOX2B*, *LINC00682*
	rs75647198	3	T	C	1.27 × 10^−14^	*PTPRG*
	rs6899869	6	G	A	5.31 × 10^−14^	*ID4*, *MBOAT1*
	rs76882751	1	A	G	8.09 × 10^−14^	*IL10*, *MAPKAPK2*
	rs17373628	6	A	G	8.44 × 10^−14^	*UTRN*, *EPM2A*
Fe	rs10461985	5	A	G	1.94 × 10^−8^	*GDNF-AS1*
	rs13435759	4	C	A	8.33 × 10^−8^	*EPHA5*
	rs12613983	2	C	A	1.59 × 10^−7^	*LINC01121*, *SIX2*
	rs1961196	14	C	T	3.84 × 10^−7^	*LINC00911*
	rs28483220	3	T	C	4.82 × 10^−7^	*MAGI1*
	rs60310655	8	G	A	6.13 × 10^−7^	*GFRA2*
	rs9644414	8	A	G	6.66 × 10^−7^	*ANGPT2*, *MCPH1*
	rs2298878	14	A	G	9.90 × 10^−7^	*HSP90AA1*
Li	rs2995976	4	C	T	1.26 × 10^−7^	*RELL1*, *PGM2*
	rs75804407	11	A	G	2.46 × 10^−7^	*GLYAT*, *CNTF*
	rs10447451	6	C	T	8.02 × 10^−7^	*SAMD5*, *STXBP5*
	rs976081	6	T	G	8.98 × 10^−7^	*ELOVL2*
Se	rs7911158	10	A	G	4.50 × 10^−11^	*CTNNA3*
	rs12407964	1	T	G	3.51 × 10^−7^	*FCAMR*
	rs5761867	22	G	A	8.13 × 10^−7^	*MN1*, *LINC01422*
	rs4541737	6	T	C	9.35 × 10^−7^	*HIST1H2BJ*, *LINC00240*
	rs72670345	4	T	C	9.35 × 10^−7^	*COL25A1*
	rs7341858	9	C	T	9.35 × 10^−7^	*COL5A1*, *RXRA*
Ti	rs1218344	10	C	A	3.61 × 10^−9^	*FRMD4A*
	rs2122084	2	G	C	1.32 × 10^−8^	*XIRP2*, *B3GALT1*
	rs10972927	9	C	A	4.08 × 10^−8^	*MELK*, *RNF38*
	rs76038730	1	G	A	4.60 × 10^−8^	*IER5*
	rs8070707	17	T	C	2.02 × 10^−7^	*PRPSAP2*
	rs35749744	13	-	G	2.35 × 10^−7^	*UFM1*
	rs75842361	21	A	G	2.95 × 10^−7^	*PRDM15*
	rs3911751	16	A	G	3.72 × 10^−7^	*CDH13*
	rs1857620	10	G	A	3.76 × 10^−7^	*ARMC4*, *MPP7*
	rs117568376	4	C	A	4.65 × 10^−7^	*RGS6*
	rs56663089	1	A	C	5.60 × 10^−7^	*PTCHD2*
V	rs2973735	5	G	A	2.44 × 10^−9^	*COL23A1*
	rs199927839	8	-	T	6.90 × 10^−8^	*COLEC10*, *TNFRSF11B*
	rs2047887	3	G	A	1.55 × 10^−7^	*CNTN6*, *LINC01266*
	rs9463728	6	T	G	1.75 × 10^−7^	*PKHD1*
	rs4525714	2	A	G	2.61 × 10^−7^	*LINC01248*, *LINC01249*
	rs78480665	12	A	C	3.03 × 10^−7^	*PPP1R12A*
	rs16873802	5	C	T	4.17 × 10^−7^	*IRX1*
	rs35376300	3	C	T	4.76 × 10^−7^	*MIR548AC*, *UBE2E1-AS1*
	rs7557937	2	G	A	5.30 × 10^−7^	*SLC4A3*, *MIR4268*
	rs16897813	8	G	A	5.61 × 10^−7^	*ZHX2*
	rs8082453	17	A	G	7.26 × 10^−7^	*AMZ2*
	rs77604578	3	A	G	8.76 × 10^−7^	*SI*, *SLITRK3*
	rs1286737	3	C	T	8.87 × 10^−7^	*RARB*

Note: dbSNP, Database of Single Nucleotide Polymorphisms; Alt, alternative allele; Ref, reference allele.

**Table A6:** Genome-wide association analysis of 21 plasma elements in a control population.

Elements	dbSNP	Chromosome	Alt	Ref	*p* Value	Associated Gene
Co	rs75430598	10	T	C	1.23 × 10^−10^	*ZMIZ1-AS1*, *LINC00595*
	rs1039968	2	C	T	1.97 × 10^−10^	*FAM117B*
	rs76944556	9	G	A	2.84 × 10^−10^	*FRMD3*, *RASEF*
	rs2242545	14	A	G	6.82 × 10^−10^	*OR4E2*, *DAD1*
	rs12588179	14	A	C	7.38 × 10^−10^	*ESRRB*
	rs4815697	20	G	A	9.65 × 10^−10^	*ADRA1D*, *PRNP*
	rs785311	2	G	A	1.32 × 10^−9^	*ASB3*, *MIR4431*
	rs35443779	9	G	A	1.73 × 10^−9^	*RXRA*
	rs13411587	2	C	T	1.89 × 10^−9^	*MYO1B*, *NABP1*
	rs34944501	19	C	T	2.09 × 10^−9^	*CCL25*, *ELAVL1*
Cr	rs11637077	15	G	T	1.92 × 10^−8^	*SLTM*, *RNF111*
	rs80242064	4	A	G	2.39 × 10^−8^	*UBE2K*
	rs13344319	19	A	G	4.98 × 10^−8^	*TTYH1*, *LAIR1*
	rs9318226	13	C	T	6.71 × 10^−8^	*KLF12*
	rs1360723	6	A	G	7.90 × 10^−8^	*EDN1*, *RNU6-48P*
	rs2701522	15	T	C	1.05 × 10^−7^	*MEIS2*, *TMCO5A*
	rs35866285	9	C	T	2.55 × 10^−7^	*INSL4*, *RLN2*
	rs79001866	2	C	T	2.80 × 10^−7^	*TEX41*
	rs2218088	3	A	C	3.28 × 10^−7^	*FHIT*
	rs9391385	6	G	A	4.19 × 10^−7^	*MTRNR2L9*, *GUSBP4*
Cu	rs647952	2	G	A	8.70 × 10^−7^	*CYP27A1*, *PRKAG3*
Fe	rs7275831	21	C	T	4.64 × 10^−9^	*ADAMTS5*, *MIR5009*
	rs11670491	19	C	G	1.79 × 10^−8^	*FGF21*, *BCAT2*
	rs73180697	8	A	G	7.60 × 10^−8^	*ERICH1-AS1*
	rs11152132	18	G	A	2.89 × 10^−7^	*GRP*, *SEC11C*
	rs116862940	2	C	T	3.28 × 10^−7^	*ANKRD30BL*
	rs11485126	1	A	C	4.72 × 10^−7^	*MIR205HG*, *PLXNA2*
	rs34872178	19	-	G	5.58 × 10^−7^	*ZNF285*
Se	rs139910427	13	-	G	6.19 × 10^−11^	*GPC6*
	rs3004034	6	T	C	9.71 × 10^−11^	*ZNF322*, *GUSBP2*
	rs79863156	18	G	A	4.33 × 10^−9^	*LINC00669*, *MIR4318*
	rs79039499	1	T	C	4.75 × 10^−9^	*CDC7*, *TGFBR3*
	rs9486924	6	T	C	4.72 × 10^−8^	*FOXO3*, *LINC00222*
	rs9510001	13	G	A	6.03 × 10^−8^	*LINC00424*, *LINC00540*
	rs3122551	1	T	G	8.18 × 10^−8^	*PPAP2B*, *MIR4422*
	rs1000321	19	G	T	8.63 × 10^−8^	*KIR3DX1*
	rs10129255	14	T	C	1.29 × 10^−7^	*MIR7641-2*
	rs28406897	22	C	T	2.33 × 10^−7^	*MICAL3*
Be	rs71598326	4	-	CTT	6.76 × 10^−7^	*LINC00613*
Cd	rs3885409	4	G	A	9.60 × 10^−7^	*CRMP1*
Mn	rs785544	1	T	C	5.48 × 10^−8^	*RGS7*
Mo	rs4722242	7	A	G	4.20 × 10^−7^	*CCDC126*, *FAM221A*
	rs11637370	15	C	T	4.74 × 10^−7^	*POLR2M*, *GCOM1*, *ALDH1A2*
	rs2493272	1	C	T	5.17 × 10^−7^	*PRDM16*
Pb	rs786402	2	C	T	1.29 × 10^−7^	*CAMKMT*
Ti	rs34320942	20	A	G	2.10 × 10^−10^	*SYNDIG1*
	rs4956073	4	A	C	3.96 × 10^−8^	*GIMD1*, *DKK2*
	rs2831693	21	A	G	5.82 × 10^−8^	*LINC00161*
	rs68050953	3	T	C	1.13 × 10^−7^	*FOXP1*
	rs9571496	13	G	A	1.17 × 10^−7^	*MIR548X2*
	rs11142949	9	G	A	1.34 × 10^−7^	*TMEM2*, *TRPM3*
	rs77190387	2	A	G	1.42 × 10^−7^	*MIR7515*, *LINC01247*, *LINC01246*
	rs12451970	17	T	G	2.36 × 10^−7^	*AXIN2*, *RGS9*, *AXIN2*
	rs116989673	9	T	G	2.88 × 10^−7^	*RFX3*, *LINC01231*
	rs34794047	15	G	A	3.19 × 10^−7^	*RGMA*

Note: dbSNP, Database of Single Nucleotide Polymorphisms; Alt, alternative allele; Ref, reference allele.

**Table A7:** Results of correlation analysis between plasma elements and metabolites in gastric cancer population.

Elements	Metabolites	Class	β (95%CI)	*p* Value
Mg	8-Isoprostaglandin E1	Lipids	−0.54 (−0.95, −0.13)	9.86 × 10^−3^
	Inosine triphosphate	Nucleosides	−0.32 (−0.58, −0.06)	1.61 × 10^−2^
	Cholic acid	Lipids	0.51 (0.07, 0.95)	2.41 × 10^−2^
	Linoleic acid	Lipids	0.27 (0.03, 0.52)	2.99 × 10^−2^
Ca	Testosterone	Lipids	−0.80 (−1.24, −0.35)	5.60 × 10^−4^
	5′-Methylthioadenosine	Nucleosides	−0.36 (−0.61, −0.11)	4.52 × 10^−3^
	8-Isoprostaglandin E1	Lipids	0.52 (0.14, 0.91)	7.95 × 10^−3^
	Uric acid	Nucleosides	0.32 (0.08, 0.55)	8.79 × 10^−3^
	(9Z,12Z)-6,8-Dihydroxy-9,12-octadecadienoic acid	Lipids	0.36 (0.09, 0.63)	1.04 × 10^−2^
	Guanosine triphosphate	Nucleosides	0.57 (0.13, 1.02)	1.25 × 10^−2^
	L-Palmitoylcarnitine	Lipids	−0.44 (−0.83, −0.06)	2.51 × 10^−2^
	Platelet-activating factor	Lipids	−0.50 (−0.94, −0.06)	2.71 × 10^−2^
	Linoleic acid	Lipids	0.25 (0.02, 0.48)	3.24 × 10^−2^
	Uridine triphosphate	Nucleosides	0.28 (0.01, 0.55)	4.52 × 10^−2^
Cr	Ornithine	Amino acids	−0.26 (−0.51, 0.00)	4.97 × 10^−2^
Fe	Alpha-Tocotrienol	Lipids	−0.50 (−0.94, −0.06)	2.52 × 10^−2^
	Phosphoribosyl-ATP	Nucleosides	−1.53 (−2.95, −0.12)	3.31 × 10^−2^
	Arachidonic acid	Lipids	1.31 (0.08, 2.55)	3.76 × 10^−2^
Co	P1,P4-Bis (5′-uridyl) tetraphosphate	Nucleosides	0.40 (0.12, 0.69)	5.54 × 10^−3^
Cu	Dipalmitoylphosphatidyl choline	Lipids	−1.93 (−2.77, −1.1)	9.72 × 10^−6^
	D- (+)-Tryptophan	Amino acids	1.88 (0.71, 3.04)	1.76 × 10^−3^
	Cytidine monophosphate	Nucleosides	−0.66 (−1.09, −0.22)	3.63 × 10^−3^
	Glutathione	peptides	−0.43 (−0.76, −0.11)	9.99 × 10^−3^
	Guanosine	Nucleosides	0.59 (0.11, 1.06)	1.58 × 10^−2^
	L-Lysine	Amino acids	−0.54 (−1.05, −0.03)	3.81 × 10^−2^
Zn	Guanosine	Nucleosides	0.73 (0.27, 1.18)	1.96 × 10^−3^
	Ornithine	Amino acids	0.68 (0.17, 1.19)	9.33 × 10^−3^
	L-Palmitoylcarnitine	Lipids	−0.48 (−0.95, −0.02)	4.13 × 10^−2^
Se	P1,P4-Bis (5′-uridyl) tetraphosphate	Nucleosides	−0.16 (−0.25, −0.06)	1.46 × 10^−3^
	8-Isoprostaglandin E1	Lipids	0.15 (0.04, 0.27)	7.67 × 10^−3^
	D- (+)-Tryptophan	Amino acids	−0.36 (−0.64, −0.09)	9.56 × 10^−3^
	Dipalmitoylphosphatidylcholine	Lipids	0.25 (0.05, 0.44)	1.40 × 10^−2^
	Caprolactam	Lipids	−0.16 (−0.30, −0.02)	2.41 × 10^−2^
	Alpha-Tocotrienol	Lipids	0.10 (0.01, 0.19)	3.08 × 10^−2^
	Cholic acid	Lipids	−0.13 (−0.25, −0.01)	3.35 × 10^−2^
	Testosterone	Lipids	−0.14 (−0.27, −0.01)	3.77 × 10^−2^
	AICAR	Nucleosides	−0.09 (−0.17, 0.00)	3.88 × 10^−2^
Mo	P1,P4-Bis (5′-uridyl) tetraphosphate	Nucleosides	0.38 (0.10, 0.67)	9.43 × 10^−3^
	11-trans-Leukotriene C4	Lipids	0.23 (0.05.0.40)	9.89 × 10^−3^
	Uridine 5′-diphosphate	Nucleosides	−0.59 (−1.07, −0.11)	1.64 × 10^−2^
	Inosine triphosphate	Nucleosides	0.26 (0.05, 0.48)	1.80 × 10^−2^
	Cytidine monophosphate	Nucleosides	−0.32 (−0.63, −0.01)	4.16 × 10^−2^
V	Methylacetoacetic acid	Lipids	−0.87 (−1.56, −0.19)	1.31 × 10^−2^
	Guanosine triphosphate	Nucleosides	0.69 (0.08, 1.29)	2.69 × 10^−2^
Mn	Taurine	Amino acids	1.01 (0.57, 1.44)	1.22 × 10^−5^
	Niacinamide	Organoheterocyclic compounds/others	−0.32 (−0.50, −0.14)	4.72 × 10^−4^
	Phosphoribosyl-ATP	Nucleosides	−1.60 (−2.62, −0.57)	2.46 × 10^−3^
	Linoleic acid	Lipids	0.34 (0.11, 0.58)	4.19 × 10^−3^
	Guanosine diphosphate mannose	Nucleosides	0.52 (0.06, 0.98)	2.62 × 10^−2^
	P1,P4-Bis (5′-uridyl) tetraphosphate	Nucleosides	0.38 (0.04, 0.71)	2.63 × 10^−2^
	Arachidonic acid	Lipids	1.00 (0.11, 1.90)	2.87 × 10^−2^
	Paraxanthine	Organoheterocyclic compounds/others	0.35 (0.02, 0.68)	3.82 × 10^−2^
Ni	Glutathione	peptides	0.40 (0.07, 0.72)	1.66 × 10^−2^
	Ornithine	Amino acids	−0.60 (−1.13, −0.08)	2.43 × 10^−2^
	L-Lysine	Amino acids	0.57 (0.07, 1.07)	2.64 × 10^−2^
	L-Palmitoylcarnitine	Lipids	−0.49 (−0.96, −0.01)	4.59 × 10^−2^
As	L-Histidinol	Amino acids	0.69 (0.21, 1.17)	5.50 × 10^−3^
	Guanosine triphosphate	Nucleosides	0.73 (0.21, 1.25)	5.99 × 10^−3^
	Adenosine monophosphate	Nucleosides	0.30 (0.06, 0.53)	1.28 × 10^−2^
	Platelet-activating factor	Lipids	−0.62 (−1.13, −0.11)	1.72 × 10^−2^
	Porphobilinogen	Lipids	−0.45 (−0.84, −0.06)	2.43 × 10^−2^
Cd	Guanosine	Nucleosides	0.70 (0.28, 1.11)	1.07 × 10^−3^
	Ornithine	Amino acids	0.62 (0.16, 1.08)	9.12 × 10^−3^
	Uric acid	Nucleosides	−0.43 (−0.76, −0.11)	9.48 × 10^−3^
	Glutathione	peptides	−0.37 (−0.66, −0.09)	1.11 × 10^−2^
	Inosine triphosphate	Nucleosides	−0.30 (−0.56, −0.03)	2.79 × 10^−2^
	L-Histidinol	Amino acids	−0.46 (−0.91, 0.00)	4.78 × 10^−2^
Pb	Guanosine triphosphate	Nucleosides	0.89 (0.39, 1.40)	6.49 × 10^−4^
	Taurine	Amino acids	0.66 (0.18, 1.15)	7.84 × 10^−3^
	Arachidonic acid	Lipids	1.01 (0.01, 2.00)	4.70 × 10^−2^
Li	L-Lysine	Amino acids	0.88 (0.36, 1.40)	1.08 × 10^−3^
	(9Z,12Z)-6,8-Dihydroxy-9,12-octadecadienoic acid	Lipids	0.48 (0.13, 0.83)	7.84 × 10^−3^
	Testosterone	Lipids	−0.66 (−1.24, −0.09)	2.46 × 10^−2^
	Guanosine triphosphate	Nucleosides	0.61 (0.04, 1.19)	3.72 × 10^−2^
Ti	Guanosine triphosphate	Nucleosides	0.74 (0.21, 1.26)	6.14 × 10^−3^
	P1,P4-Bis (5′-uridyl) tetraphosphate	Nucleosides	0.50 (0.12, 0.88)	1.07 × 10^−2^
	Glycoursodeoxycholic acid	Lipids	−0.28 (−0.50, −0.05)	1.66 × 10^−2^
	Ornithine	Amino acids	−0.56 (−1.06, −0.06)	2.77 × 10^−2^
Sr	Glycoursodeoxycholic acid	Lipids	−0.37 (−0.63, −0.1)	6.49 × 10^−3^
	Epsilon-Caprolactam	Lipids	0.83 (0.19, 1.47)	1.11 × 10^−2^
	Guanosine diphosphate mannose	Nucleosides	0.66 (0.06, 1.27)	3.19 × 10^−2^
	Adenosine monophosphate	Nucleosides	0.28 (0.01, 0.56)	4.45 × 10^−2^
	Platelet-activating factor	Lipids	−0.60 (−1.20, −0.01)	4.77 × 10^−2^
Sb	LBF	peptides	0.59 (0.22, 0.96)	1.55 × 10^−2^
	Cholic acid	Lipids	−0.36 (−0.64, −0.08)	2.15 × 10^−3^
	Alpha-Tocotrienol	Lipids	0.55 (0.11, 0.99)	1.15 × 10^−2^
	Arachidonic acid	Lipids	0.89 (0.10, 1.68)	2.66 × 10^−2^
Tl	Glutathione	peptides	−0.51 (−0.83, −0.18)	2.37 × 10^−3^
	8-Isoprostaglandin E1	Lipids	−0.66 (−1.13, −0.18)	6.96 × 10^−3^
	Alpha-Tocotrienol	Lipids	−0.46 (−0.85, −0.08)	1.90 × 10^−2^
	Niacinamide	Organoheterocyclic compounds/others	−0.25 (−0.47, −0.04)	2.06 × 10^−2^
	Guanosine	Nucleosides	0.54 (0.07, 1.01)	2.44 × 10^−2^
	Linoleic acid	Lipids	0.32 (0.03, 0.60)	2.95 × 10^−2^
	P1,P4-Bis (5′-uridyl) tetraphosphate	Nucleosides	0.43 (0.02, 0.83)	3.91 × 10^−2^

**Table A8:** Results of correlation analysis between plasma elements and metabolites in control population.

Elements	Metabolites	Class	β (95%CI)	*p* Value
Mg	Uridine triphosphate	Nucleosides	−0.35 (−0.66, −0.04)	2.58 × 10^−2^
	Porphobilinogen	Lipids	−0.47 (−0.91, −0.02)	4.03 × 10^−2^
	Phosphoribosyl-ATP	Nucleosides	1.67 (0.06.3.27)	4.16 × 10^−2^
	Guanosine	Nucleosides	−0.46 (−0.92, 0.00)	4.88 × 10^−2^
Ca	Arachidonic acid	Lipids	−0.58 (−1.09, −0.06)	3.01 × 10^−2^
Cr	Uridine triphosphate	Nucleosides	0.59 (0.20, 0.97)	2.98 × 10^−3^
	Arachidonic acid	Lipids	−0.86 (−1.43, −0.29)	3.50 × 10^−3^
	Phosphoribosyl-ATP	Nucleosides	−2.65 (−4.64, −0.66)	9.53 × 10^−3^
	Uric acid	Nucleosides	0.47 (0.09, 0.84)	1.46 × 10^−2^
Fe	AICAR	Nucleosides	0.53 (0.13, 0.93)	1.00 × 10^−2^
	Cer (d18:0/12:0)	Lipids	−0.82 (−1.49, −0.15)	1.73 × 10^−2^
	Guanosine	Nucleosides	−0.44 (−0.85, −0.03)	3.74 × 10^−2^
	Adenosine monophosphate	Nucleosides	−0.25 (−0.50, −0.01)	4.30 × 10^−2^
Co	3-Carboxy-1-hydroxypropylthiamine-diphosphate	Organoheterocyclic compounds/others	−0.50 (−0.86, −0.13)	7.57 × 10^−3^
	Dihydrobiopterin	Organoheterocyclic compounds/others	0.41 (0.04, 0.78)	2.90 × 10^−2^
	Adenosine monophosphate	Nucleosides	0.31 (0.01, 0.60)	3.95 × 10^−2^
Cu	Uridine 5′-diphosphate	Nucleosides	−0.69 (−1.28, −0.10)	2.21 × 10^−2^
	Phosphoribosyl-ATP	Nucleosides	1.50 (0.12, 2.87)	3.27 × 10^−2^
Zn	L-Histidinol	Amino acids	0.59 (0.10, 1.08)	1.75 × 10^−2^
	Uric acid	Nucleosides	0.35 (0.04, 0.65)	2.76 × 10^−2^
Se	(9Z,12Z)-6,8-Dihydroxy-9,12-octadecadienoic acid	Lipids	0.81 (0.16, 1.47)	1.49 × 10^−2^
	Cholic acid	Lipids	−1.09 (−2.05, −0.12)	2.73 × 10^−2^
Mo	Cholic acid	Lipids	1.12 (0.39, 1.86)	2.99 × 10^−3^
	Linoleic acid	Lipids	−0.48 (−0.81, −0.15)	5.04 × 10^−3^
	N-Acetylmuramate	Organoheterocyclic compounds/others	0.49 (0.13, 0.86)	8.73 × 10^−3^
	Uridine triphosphate	Nucleosides	0.44 (0.11, 0.77)	1.01 × 10^−2^
	Ornithine	Amino acids	1.03 (0.22, 1.84)	1.33 × 10^−2^
	Testosterone	Lipids	−0.68 (−1.24, −0.13)	1.66 × 10^−2^
	Arachidonic acid	Lipids	−0.59 (−1.07, −0.10)	1.93 × 10^−2^
	FADH	Nucleosides	−0.59 (−1.11, −0.06)	2.95 × 10^−2^
	Uric acid	Nucleosides	0.34 (0.02, 0.66)	3.66 × 10^−2^
V	Phosphoribosyl-ATP	Nucleosides	−1.64 (−3.06, −0.21)	2.47 × 10^−2^
Ni	Leukotriene C4	Lipids	0.34 (0.05, 0.63)	2.07 × 10^−2^
	L-Palmitoylcarnitine	Lipids	0.41 (0.03, 0.79)	3.56 × 10^−2^
	Phosphoribosyl-ATP	Nucleosides	−1.56 (−3.07, −0.04)	4.42 × 10^−2^
As	Guanosine	Nucleosides	−0.62 (−1.09, −0.16)	9.28 × 10^−3^
	Uric acid	Nucleosides	−0.19 (−0.34, −0.03)	1.81 × 10^−2^
	AICAR	Nucleosides	0.46 (0.01, 0.91)	4.63 × 10^−2^
	3-Carboxy-1-hydroxypropylthiamine-diphosphate	Organoheterocyclic compounds/others	0.34 (0.00, 0.69)	4.77 × 10^−2^
Cd	11-trans-Leukotriene C4	Lipids	0.34 (0.07, 0.61)	1.50 × 10^−2^
	L-Lysine	Amino acids	−0.65 (−1.27, −0.02)	4.21 × 10^−2^
Pb	Glutathione	peptides	0.39 (0.07, 0.70)	1.60 × 10^−2^
	Linoleic acid	Lipids	−0.31 (−0.57, −0.04)	2.26 × 10^−2^
	Dipalmitoylphosphatidyl choline	Lipids	−0.30 (−0.57, −0.03)	2.77 × 10^−2^
Li	Proline	Amino acids	0.29 (0.01, 0.58)	4.55 × 10^−2^
Sr	Arachidonic acid	Lipids	−1.06 (−1.98, −0.15)	2.34 × 10^−2^
Sb	3-Carboxy-1-hydroxypropylthiamine-diphosphate	Organoheterocyclic compounds/others	0.59 (0.25, 0.94)	8.39 × 10^−4^
	SM (d18:1/16:0)	Lipids	−0.63 (−1.11, −0.15)	1.01 × 10^−2^
	Sulfate	Organoheterocyclic compounds/others	0.92 (0.21, 1.62)	1.15 × 10^−2^
	Methylacetoacetic acid	Organoheterocyclic compounds/others	−0.76 (−1.41, −0.12)	2.06 × 10^−2^
	Arachidonic acid	Lipids	0.49 (0.02, 0.96)	3.99 × 10^−2^

**Table A9:** Genome-wide association analysis of 57 plasma metabolites in gastric cancer population.

Metabolites	dbSNP	Chromo Some	Alt	Ref	*p* Value	Associated Gene
Proline	rs4607880	1	G	A	3.10 × 10^−7^	*MAPKAPK2*
	rs992923	5	C	A	5.87 × 10^−7^	*MIR4280*
L-Palmitoylcarnitine	rs1542829	3	A	G	5.81 × 10^−7^	*COL6A5*
Glycoursodeoxycholic acid	rs2827509	21	C	A	6.74 × 10^−7^	*LINC00308*
DL-Dipalmitoylphosphatidylcholine	rs9391014	6	A	C	3.90 × 10^−7^	*HACE1*, *GRIK2*
Arachidonic acid	rs978935	21	T	G	3.89× 10^−8^	*PCP4*, *DSCAM*
	rs75551965	8	G	T	2.22 × 10^−7^	*EIF4EBP1*, *ASH2L*
	rs76731126	7	C	G	5.52 × 10^−7^	*KCND2*, *ANKRD7*
Guanosine diphosphate mannose	rs79300593	6	G	A	3.36 × 10^−11^	*HACE1*, *GRIK2*
	rs2918299	19	T	C	2.28 × 10^−10^	*ACTL9*, *ADAMTS10*
	rs2164983	19	A	C	2.28 × 10^−10^	*ACTL9*, *ADAMTS10*
	rs9876082	3	A	G	4.12 × 10^−10^	*PRKCI*
	rs2229259	19	T	C	5.22 × 10^−10^	*ECH1*
	rs9895829	17	G	A	5.58 × 10^−10^	*TP53*
	rs1978980	7	C	G	8.74 × 10^−10^	*ACTR3B*
	rs7788967	7	A	G	9.71 × 10^−10^	*CREB3L2*
	rs76212525	3	C	G	1.07 × 10^−9^	*CRBN*
	rs60907119	17	C	T	1.64 × 10^−9^	*DNAH9*
	rs12051736	17	C	T	2.91 × 10^−9^	*OR3A1*, *OR3A2*
	rs247616	16	T	C	3.19 × 10^−9^	*HERPUD1*, *CETP*
	rs139871882	1	T	C	4.75 × 10^−9^	*TRIM67*
	rs7382159	6	C	A	4.75 × 10^−9^	*ZBED9*
	rs7771146	6	G	A	4.89 × 10^−9^	*PERP*
	rs76473604	2	G	C	4.94 × 10^−9^	*SPOPL*, *HNMT*
	rs3764339	16	T	C	5.20 × 10^−9^	*HYDIN*
	rs77422016	8	C	A	7.63 × 10^−9^	*SLC39A4*
	rs12821783	12	T	C	1.02 × 10^−8^	*PRICKLE1*
	rs7034951	9	T	C	1.14 × 10^−8^	*KLF4*, *ACTL7B*
	rs4696423	4	G	C	1.36 × 10^−8^	*TRIM2*
	rs62451896	7	A	G	1.38 × 10^−8^	*POM121L12*
	rs10152068	14	G	A	1.46 × 10^−8^	*RPGRIP1*
	rs41532344	11	T	C	1.99 × 10^−8^	*MUC2*
	rs2303150	19	T	C	2.64 × 10^−8^	*TPM4*
	rs9461366	6	A	G	3.14 × 10^−8^	*VN1R10P*
	rs35301767	12	A	G	3.85 × 10^−8^	*NEU4*
	rs116942374	11	G	T	4.03 × 10^−8^	*SYTL2*
	rs1409696	9	T	C	5.25 × 10^−8^	*TMEM215*
	rs13380367	15	A	C	6.54 × 10^−8^	*FMN1*
	rs11210359	1	A	G	6.57 × 10^−8^	*LRRIQ3*, *LINC01360*
	rs16952946	15	C	T	6.94 × 10^−8^	*SLC24A5*
	rs9486801	6	A	G	1.23 × 10^−7^	*OSTM1*, *NR2E1*
	rs17159830	5	T	C	1.30 × 10^−7^	*EFNA5*
	rs6079582	20	T	C	1.82 × 10^−7^	*MACROD2*
	rs16892645	4	T	C	1.98 × 10^−7^	*FGFBP1*
	rs57365465	10	G	-	2.46 × 10^−7^	*ST8SIA6*, *PTPLA*
	rs7661304	4	G	A	2.51 × 10^−7^	*FAT1*
	rs4941088	18	G	T	3.13 × 10^−7^	*RNF152*, *PIGN*
	rs11141592	9	G	A	3.33 × 10^−7^	*GAS1*, *ZCCHC6*
	rs6045843	20	C	T	3.61 × 10^−7^	*SLC24A3*
	rs11018025	10	A	G	4.49 × 10^−7^	*TCERG1L*, *LINC01164*
	rs6700888	1	A	C	4.52 × 10^−7^	*RSG1*
	rs254893	5	A	G	5.04 × 10^−7^	*FGF18*, *SMIM23*
	rs1424241	16	A	G	5.42 × 10^−7^	*TXNL4B*, *DHODH*
	rs3826910	19	C	T	5.86 × 10^−7^	*MARK4*
	rs1355223	11	G	A	6.91 × 10^−7^	*EHF*, *APIP*
	rs474166	8	G	A	7.16 × 10^−7^	*MTDH*
	rs10069479	5	G	A	7.72 × 10^−7^	*LINC00992*
	rs1119098	6	G	C	8.95 × 10^−7^	*GABBR1*
	rs12665186	6	G	A	9.31 × 10^−7^	*HCG9*, *ZNRD1-AS1*
Phosphoribosyl-ATP	rs978935	21	T	G	1.19× 10^−8^	*PCP4*, *DSCAM*

Note: dbSNP, Database of Single Nucleotide Polymorphisms; Alt, alternative allele; Ref, reference allele.

**Table A10:** Genome-wide association analysis of 57 plasma metabolites in control population.

Metabolites	dbSNP	Chromo Some	Alt	Ref	*p* Value	Associated Gene
Proline	rs510795	6	C	T	3.83 × 10^−7^	*RNF217*
	rs9811803	3	G	A	5.51 × 10^−7^	*LYZL4*, *CCK*
	rs16907800	9	A	G	6.19 × 10^−7^	*ZNF618*, *RGS3*
	rs62062187	17	A	G	7.08 × 10^−7^	*TMEM132E*, *CCT6B*
	rs7204252	16	T	C	7.95 × 10^−7^	*CDIPT*, *MVP*
	rs16879060	8	T	G	8.79 × 10^−7^	*NRG1*
Methylacetoacetic acid	rs17400041	9	C	G	9.21 × 10^−7^	*RASEF*, *FRMD3*
Niacinamide	rs1999299	21	T	C	3.79 × 10^−7^	*NCAM2*, *LINC00317*
	rs36000110	1	G	A	9.35 × 10^−7^	*SNX7*
	rs79107046	4	-	G	9.73 × 10^−7^	*GALC*
Ornithine	rs715275	21	C	G	6.53 × 10^−7^	*LINC00159*, *MIS18A*
	rs58268150	8	A	G	8.65 × 10^−7^	*GFRA2*
Porphobilinogen	rs17139180	7	G	A	2.49 × 10^−7^	*HDAC9*
Alpha-Tocotrienol	rs75235779	12	A	G	3.22 × 10^−8^	*MED13L*, *TBX3*
	rs479229	22	G	A	2.35 × 10^−7^	*MN1*, *LINC01422*
	rs2956091	11	G	A	2.54 × 10^−7^	*APIP*
	rs76285119	16	G	A	2.65 × 10^−7^	*CHP2*, *PRKCB*
	rs12135591	1	A	G	2.97 × 10^−7^	*LPHN2*
	rs7116460	11	T	C	4.99 × 10^−7^	*ZBTB16*
	rs11018868	11	C	T	5.45 × 10^−7^	*NAALAD2*, *UBTFL1*
Glycoursodeoxycholic acid	rs78874851	1	C	T	9.52 × 10^−8^	*RYR2*, *MT1HL1*
	rs10513821	3	G	A	2.77 × 10^−7^	*BCL6*, *LPP-AS2*
	rs58243993	11	C	T	7.04 × 10^−7^	*GALNT18*
Uridine triphosphate	rs77647216	17	C	T	8.84 × 10^−7^	*CACNG4*, *CACNG5*
DL-Dipalmitoylphosphatidylcholine	rs4517394	1	G	A	7.36 × 10^−7^	*SDC3*, *PUM1*
	rs17827063	14	G	A	8.70 × 10^−7^	*FUT8*, *LINC00238*
Platelet-activating factor	rs774745	3	C	T	2.05 × 10^−7^	*ABHD10*
3-Carboxy-1-hydroxypropylthiamine-diphosphate	rs12612311	2	G	A	3.71 × 10^−8^	*STK39*, *CERS6*
	rs325724	3	G	A	9.07 × 10^−8^	*MBNL1-AS1*, *AADACL2-AS1*
	rs12487740	3	G	T	1.16 × 10^−7^	*GAP43*
	rs11056455	12	C	T	1.23 × 10^−7^	*PTPRO*
	rs2064530	6	A	C	1.31 × 10^−7^	*QKI*
	rs10735020	12	C	T	1.64 × 10^−7^	*TSPAN9*, *PRMT8*
	rs10497464	2	C	T	3.45 × 10^−7^	*HNRNPA3*
	rs146622680	19	C	T	3.59 × 10^−7^	*HAVCR1P1*, *LINC00662*
	rs13276609	8	A	C	4.25 × 10^−7^	*KCNV1*
	rs6597390	6	A	C	4.99 × 10^−7^	*TFAP2A*
	rs35513383	18	-	A	7.37 × 10^−7^	*PTPRM*
	rs76806262	14	C	T	7.86 × 10^−7^	*NRXN3*
	rs8615	20	T	C	8.95 × 10^−7^	*NAPB*
	rs1047636	11	T	C	9.06 × 10^−7^	*AASDHPPT*, *KBTBD3*
	rs11726114	4	A	G	9.17 × 10^−7^	*MIR548AG1*
	rs4664353	2	G	A	9.97 × 10^−7^	*RBMS1*, *TANK*
LBF	rs198472	11	G	C	4.60 × 10^−7^	*MYRF*
	rs174541	11	C	T	2.27 × 10^−10^	*MYRF*, *TMEM258*
	rs174538	11	A	G	3.78 × 10^−10^	*MYRF*, *TMEM258*
	rs174537	11	T	G	4.05 × 10^−10^	*TMEM258*
	rs102274	11	C	T	4.05 × 10^−10^	*TMEM258*
	rs174548	11	G	C	4.72 × 10^−10^	*TMEM258*
	rs174549	11	A	G	9.21 × 10^−10^	*FEN1*, *FADS2*
	rs174545	11	G	C	2.64 × 10^−8^	*FADS1*, *FADS2*
	rs2851682	11	G	A	3.64 × 10^−8^	*FADS1*, *FADS2*
	rs102275	11	C	T	4.61 × 10^−8^	*FADS1*, *FADS2*
	rs2072114	11	G	A	7.62 × 10^−8^	*FADS1*, *FADS2*
	rs174528	11	C	T	1.11 × 10^−7^	*FADS1*, *FADS2*
	rs174547	11	C	T	1.46 × 10^−7^	*FADS1*, *FADS2*
	rs174601	11	T	C	1.62 × 10^−7^	*FADS2*
	rs4246215	11	T	G	1.66 × 10^−7^	*FADS2*
	rs174555	11	C	T	1.69 × 10^−7^	*FADS2*
	rs2845573	11	G	A	1.87 × 10^−7^	*FADS2*
8-Isoprostaglandin E1	rs774745	3	C	T	1.13 × 10^−7^	*ABHD10*
Guanosine diphosphate mannose	rs10513821	3	G	A	2.17 × 10^−7^	*BCL6*, *LPP-AS2*
	rs1444292	15	T	C	7.48 × 10^−7^	*KLHL25*
FADH	rs35096828	5	C	T	6.35 × 10^−7^	*JADE2*
Cer (d18:0/12:0)	rs10025052	4	T	C	2.78 × 10^−8^	*TBCK*
	rs774745	3	C	T	2.37 × 10^−7^	*ABHD10*
	rs4664353	2	G	A	8.68 × 10^−7^	*RBMS1*, *TANK*

Note: dbSNP, Database of Single Nucleotide Polymorphisms; Alt, alternative allele; Ref, reference allele.
