# Supplementary material for: Integrated Elementomics–Genomics–Metabolomics Analysis Reveals Plasma Biomarker Networks and Diagnostic Potential for Gastric Cancer
Source: Metabolites. 2026 Jul 10;16(7):487. doi: 10.3390/metabo16070487 (PMC13414432; doi:10.3390/metabo16070487)
Supplement: Supplementary file 1 [file metabolites-16-00487-s001.zip › Supplementary File S1. Detailed experimental procedures for peripheral blood genotyping and non-targeted plasma metabolite analysis..pdf]

Peripheral Blood Genotyping

DNA amplification

(1)Preparations for DNA Amplification

1) Preparation of Instruments and Consumables

No.	Instrument	No.	Consumables	Quantity
1	Preheat the hybridization oven to 37°C	1	15 mL centrifuge tubes	1
2	Turn on the room-temperature centrifuge	2	50mL centrifuge tubes	2
3	Set the timer	3	Clean reagent trays	2
4	12-channel pipette: 20, 200, 1200μL	4	20, 200, and 1200 μL tips	1box

2) Reagent Preparation: Axiom 2.0 Module 1

No.	Reagent Name	Processing
1	Axiom2.0 Denat Soln 10x	Shake, centrifuge, and allow to reach room temperature
2	Axiom2.0 Neutral Soln	Shake, centrifuge, and allow to reach room temperature
3	Axiom2.0 Amp Soln	Shake, centrifuge, and allow to reach room temperature
4	Axiom Water	Shake, centrifuge, and allow to reach room temperature
5	Axiom2.0 Amp Enzvme	Store at -20°C prior to use; gently tap three times before use

Note: All reagents must be allowed to reach room temperature before use, and the Axiom 2.0 Amp Solution must be thoroughly mixed.

3)Preparation of DNA Samples for Testing

① Dilute genomic DNA to 10 ng/μL in 20 μL volumes and dispense into a 96-well deep-well plate.

② Allow the genomic DNA to reach room temperature on the laboratory bench and keep it at room temperature until use.

(2) Pre-amplification Denaturation (DS)

Prepare the Denaturation Master Mix (DS)

Reagent (DS)	1 sample	1×Plate (96 sample)
Axiom 2.0 Denat Soln 10X	2μL	400μL
Axiom Water	18μL	3.6mL
Total	20μL	4mL

Note: Dispense 100 μL per well into a 24-well strip; add 20 μL of DS to each sample well; add the reagent to the bottom of the sample tube without pipetting up; seal the strip, shake to mix, start the timer for 10 minutes, and centrifuge at room temperature; after incubating at room temperature for 10 minutes, proceed immediately to the next step.

(3) Neutralization Prior to DNA Amplification (NS)

1) Pour Axiom 2.0 Neutral Soln (NS) into a clean reagent well.

2) Add 130 μL of Neutral Soln (NS) to each sample, seal the plate, vortex, and centrifuge.

(4) DNA Amplification (AM)

1) Prepare the Amplification Master Mix (AM)

Reagents（AM）	1 sample	1×Plate
Axiom2.0 Amp Soln	225μL	26mL
Axiom2.0 Amp Enzyme	5μL	578μL
Total Volume	230μL	26.58mL

Note: Vortex the AM thoroughly to mix, then invert the tube to ensure complete mixing.

2) Add 230 μL of AM to each sample well, keeping the tip against the wall of the well and allowing the solution to flow in slowly; do not aspirate.

3) Wipe any liquid from the surface of the plate, seal it tightly with the cover, shake twice, and centrifuge at 1,000 rpm for 1 minute at room temperature.

4) Gently place the sample plate containing AM into a 37°C hybridization oven and incubate for 23 ± 1 hours.

DNA Fragmentation and Precipitation Following Amplification

(1) Preparation for DNA Fragmentation

1）Preparation of Instruments and Consumables

No.	Instrument	No.	Consumables	Quantity
1	Preheat hybridization oven to 37°C	1	15mL centrifuge tube	1
2	Preheat hybridization oven to 65°C	2	50 centrifuge tubemL	2
3	Room-temperature centrifuge and agar	3	Clean reagent tray	2
4	Timer	4	20, 200, and 1200 μL pipette tips	1 box each

No.	Instrument	No.	Consumables	Quantity
5	12-channel pipette (20, 200, and 1200			

2)Reagent Preparation: Axiom2.0 Moudle 2-1; Axiom2.0 Moudle 2-2

No.	Reagent Name	Moudle	Processing
1	Axiom 10x Fraq Buffer	2-1	Thaw at room temperature, vortex, centrifuge, place on ice
2	Axiom Fraq Diluent	2-2	Vortex, centrifuge, place on ice
3	Axiom Fraq Enzyme	2-1	Gently tap 3 times in a -20°C freezer, store at -20°C
4	Axiom Fraq Rxn Stop	2-2	Vortex, centrifuge, allow to reach room temperature
5	Precip Soln 1	2-2	Vortex, centrifuge, allow to reach room temperature
6	Precip Soln 2	2-1	Thaw at room temperature, vortex, centrifuge, leave at room temperature
7	Isopropyl alcohol	NA	Leave at room temperature

(2) Terminate the amplification reaction

1) Stop the amplification reaction and record the amplification duration; the typical amplification time is 23±1 hours.

2) Remove the sample plate from the 37°C hybridization chamber and place it in the 65°C hybridization oven for 20 minutes.

3) Remove the sample plate from the 65°C hybridization oven and place it in the 37°C hybridization chamber for 45 minutes.

(3) DNA fragmentation

1) Prepare the Fragmentation Master Mix (FM)

Reagents（FM）	1 sample	1×Plate
Axiom 10X Frag Buffer	45.7μL	6mL
Axiom Frag Diluent	10.3μL	1.35mL
Axiom Frag Enzyme	1μL	131μL
Total	57μL	7.48mL

Note: Begin preparing the FM 5 minutes before the end of the 37°C incubation. Add the Axiom Frag Enzyme at the end of the 37°C incubation. After mixing by shaking, pour the FM into a clean reagent well and leave it at room temperature. Note: This step is sensitive to temperature changes; perform the procedure quickly to minimize the time the sample plate spends outside the 37°C hybridization oven.

2) Remove the sample plate from the 37°C hybridization oven and place it on the bench.

3) Add 57 μL of FM to each sample well.

4) Secure the sealing film and shake the sample plate to mix the contents.

5) Start a 30-minute timer.

6) Briefly centrifuge the sample plate at room temperature, then return it to the 37°C hybridization oven.

Note: This step requires precise timing. Be sure to keep track of the time until the end of the 30-minute incubation period, and ensure that you begin the next step to stop the reaction immediately after the timer goes off.

(4) DNA Fragmentation Termination Reaction

1) A few minutes in advance, pour the Axiom Frag Rxn Stop reagent into a clean reagent well and leave it at room temperature.

2) A few seconds before the end of the incubation, remove the sample plate from the hybridization oven and place it on the bench.

3) At the end of 30 minutes (when the timer alerts you), immediately add 19 μL of Axiom Frag Rxn Stop to the sample plate.

4) Seal the plate with the sealing film, shake to mix the contents, centrifuge at room temperature, and set aside.

(5) Isopropyl alcohol precipitation

1）Precipitation Master Mix(PM)

Reagents (PM)	1 sample	1×Plate
Axiom Precip Soln 1	238μL	26mL
Axiom Precip Soln 2	2μL	218μL
Total Volume	240μL	26.22mL

2) Add 240 μL of Precipitation Master Mix (PM) to each sample, seal the well, vortex, and centrifuge.

3) Add 600 μL of isopropanol to each sample and pipette up and down several times to ensure thorough mixing.

4) After sealing the well, transfer the samples to -20°C and incubate for 16–24 hours.

Drying, Resuspension, and Quality Control of Fragmented DNA

(1) Preparations

1）Preparation of Instruments and Consumables

No.	Instrument	No.	Consumables	Quantity
1	Preheat the hybridization oven to 37℃	1	15mL centrifuge tubes	1
2	4℃ centrifuge and agar plates	2	50mL centrifuge tubes	2
3	Timer	3	Clean reagent tray	2
4	12-channel pipette (20, 200, 1200 μL)	4	Tips: 20, 200, 1200μL	1 box each

2）Reagent Preparation

NO.	Reagent Name	Module	Procedure
1	Axiom Hvb Buffer	2-1	Shake, leave at room temperature
2	Axiom Hvb Soln 1	2-1	Thaw, vortex, centrifuge, leave at room temperature
3	Axiom Hvb Soln 2	2-2	Vortex, centrifuge, leave at room temperature
4	Axiom Resusp Buffer	2-2	Thaw to room temperature (1 hour)

(2) Drying

1) Remove the sample plate from the -20℃ freezer and place it in a pre-chilled 4℃ centrifuge. Centrifuge at 3200 ×g for 40 minutes.

2) Discard the supernatant and invert the sample plate onto absorbent paper for 5 minutes. (Carefully observe the sediment at the bottom of the tubes.)

3) Turn the sample plate right side up and place it in a 37℃ hybridization oven to dry. Drying time is 10–20 minutes; after 10 minutes, adjust the duration based on the actual dryness of the sample plate.

4) If no further experiments are planned, the dried samples can be stored at -20℃ after sealing tightly with a sealing film.

(3) Resuspension

1) If the sample plate is stored at -20℃, allow the samples to stand at room temperature

for 1.5 hours before resuspension.

2) Add 35 μL of Resuspension Buffer to each sample well.

3) Dissolve using a shaker for 20 minutes.

4) Prepare the Hybridization Master Mix.

Reagents（Hvb）	1 sample	1×Plate
Axiom Hvb Buffer	70.5μL	7.8mL
Axiom Hvb Soln 1	0.5μL	55.6μL
Axiom Hvb Soln 2	9.0μL	1.0mL
Total Volume	80.0μL	8.86mL

5) Transfer all dissolved samples to a new 96-well plate, add 80 μL of hybridization buffer to each well, seal the plate, shake, and centrifuge

(4) Electrophoresis Quality Control

1) Add 33 μL of sterile water and 3 μL of the sample to be hybridized to a new 96-well plate, seal with a film, shake, and centrifuge; label this as the "QC Diln" plate.

2) Add 10 μL of the sample from the "QC Diln" plate and 90 μL of sterile water to a 96-well optical plate; label this as the "OD" plate.

3) Measure the concentration of the "OD" plate sample using a microplate reader.

4) Pipette 3 μL of the "QC Diln" plate sample for electrophoresis using a 4% gel.

5) Evaluate the quality control results based on the electrophoresis pattern. If any samples fail the test, record the lane number of the non-conforming sample.

(5) Mutagenic Hybridization

Preparation of instruments and consumables for the mutagenic hybridization step:

NO.	Equipment	NO.	Consumables	Quantity
1	Gene Titan (air compressor)	1	Module 3	1
2	Preheat metal blocks in the 48-well hybridization oven	2	10、200μL tips	1
3	PCR instrument	3	Preheat chip at room temperature for 25 minutes	NA
4	12-channel 200-μL pipette			

1) Launch the GTGS software, create a project and sample plate in the designated folder,

and enter the information for the samples to be hybridized.

2) Once the chip has finished preheating, open the packaging and scan the barcode into the GeneTitan MC system.

3) After confirming that the GeneTitan system is ready to hybridize the new chip, start the denaturation program.

4) Denaturation program: 95℃ for 10 min; 48℃ for 3 min; 48℃ indefinitely

Note: If the samples to be hybridized are stored at -20℃, thaw them at room temperature first and allow them to reach room temperature.

5) Place the Hyb Tray and the denatured samples on the metal block preheated to 48℃ for processing.

6) Dispense 105 μL of denatured sample into the Hyb Tray, taking care to avoid creating air bubbles.

7) Following the prompts on the GeneTitan MC system, place the Hyb Tray and the chip (with the lid removed but the blue holder retained) in the designated positions.

8) Hybridization time: 23.5–24 hours.

9) Record the chip's Bacode and its position in the GeneTitan hybridizer (left or right).

Chip Cleaning, Staining, and Scanning

(1) Preparation of Instruments and Consumables

NO.	Instrument	NO.	Consumables	Quantity
1	Gene Titan MC Hybridization (Normal)	1	15/50mL centrifuge tubes	3-1
2	Air Compressor (Power On)	2	Module 3 Antistatic Consumables	1set

(2) Reagent Preparation: Axiom2.0 Moudle 4-1；Axiom2.0 Moudle 4-2

NO.	Reagent Name	Moudle	Procedure
1	Axiom Liqate Enzyme	4-1	Store at -20℃ until use
2	Other reagents (protect from light)	4-1	Thaw, vortex, centrifuge, and place on ice
3	Axiom Wash A	4-2	Vortex, centrifuge, store at room temperature

NO.	Reagent Name	Moudle	Procedure
4	Axiom Liqate Soln 2	4-2	Vortex, centrifuge, store at room temperature
5	Axiom Hold Buffer	4-2	Vortex, centrifuge, store at room temperature
6	Other reagents (protect from light)	4-2	Vortex, centrifuge, store at room temperature

A. Preparation of the S1 System

Reagent（S1）	1 sample	1×Plate
Axiom Wash A	201.6μL	22.2mL
Axiom Stain Buffer	4.2μL	463μL
Axiom Stain 1-A	2.1μL	231μL
Axiom Stain 1-B	2.1μL	231μL
Total	210μL	23.13mL

B. Preparation of the S2 System

Reagent（S2）	1 sample	1×Plate
Axiom Wash A	100.8μL	11.1mL
Axiom Stain Buffer	2.1μL	231μL
Axiom Stain 2-A	1.05μL	115.6μL
Axiom Stain 2-B	1.05μL	115.6μL
Total	105μL	11.56mL

C. Preparation of the FIX System

Reagent（FIX）	1 sample	1×Plate
Axiom Water	93.19μL	10.3mL
Axiom Stabilize Diluent	10.50μL	1.16mL
Axiom Stabilize Soln	1.31μL	144.8μL
Total	105μL	11.61mL

D. Preparation of the Lig Mix System

Reagent（Liq-M1）	1 sample	1×Plate（96 samples）
Axiom Liqate Buffer	66.15μL	7.3mL

Axiom Liqate Buffer	13.12μL	1.45mL
Axiom Liqate Soln 2	3.15μL	348μL
Total	82.42μL	9.10mL

Note: Thoroughly mix Lig-M1 using a shaker before proceeding with the preparation of the Lig Mix.

Reagent（Liq Mix）	1 sample	1×Plate（96 samples）
Liq-M1	82.42μL	9.10mL
Axiom Prob Mix 1	10.5μL	1.16mL
Axiom Prob Mix 2	10.5μL	1.16mL
Axiom Liqate Enzyme	1.58μL	174.4μL
Total	105μL	11.58mL

Note: Invert the Lig-Mix several times to ensure thorough mixing; do not shake vigorously using a shaker.

(3) Dispensing of washing solutions

1) Dispense 2 aliquots of Stain 1 Master Mix per sample, 105 μL each; dispense 1 aliquot of Stain 2 Master Mix and Stabilization Master Mix per sample, 105 μL each.

2) Add 150 μL of Holding Buffer to each well of the scan tray.

(4) Precautions for Washing and Scanning

1) Each plate must be labeled, and labels must be placed only on the side with the “affy” label.

2) Add the various staining reagents in sequence as prompted by the software.

3) Place the plate and lid into the GeneTitan MC, ensuring that the notched side is correctly aligned.

4) When placing the Scan Tray into the GeneTitan MC, remove the black base.

5) After scanning is complete, eject the chip from the system, place it on the black stand, seal it in packaging, and store it at 95℃ . If scanning is incomplete, rescan and record the rescan location.

Non-targeted plasma metabolite analysis

Chromatographic conditions

Chromatography column: Agilent SB-C18 column(2.1x50mm，1.8μm)；

Column temperature: Maintain at 37℃; injection volume: 20 μL per injection;

Mobile phase: A = ultrapure water (0.1% formic acid), B = pure acetonitrile. Prior to the experiment, place the mobile phase in a KQ-250E CNC ultrasonic cleaner for ultrasonic cleaning to remove any air bubbles, which could affect the experimental results; Linear gradient elution for samples: Flow rate 0.25 mL/min; equilibrate for 30 minutes before each batch injection; elute completely from A:B (98:2) to A:B (0:100) within 30 minutes; elute with ultrapure water after each sample, as follows: Samples:

Time (min)	Flow Rate(mL/min)	A(%)	B(%)	Pressure(bar)
0.00	0.25	98.0	2.0	400.00
9.00	0.25	40.0	60.0	400.00
18.00	0.25	40.0	60.0	400.00
20.00	0.25	0.0	100.0	400.00
30.00	0.25	0.0	100.0	400.00

Ultrapure water:

Time (min)	Flow Rate(mL/min)	A(%)	B(%)	Pressure(bar)
7.00	0.25	2.0	98.0	400.00
14.00	0.25	98.0	2.0	400.00

Mass Spectrometry Conditions

Equipped with a TurboIonspray (ESI) ion source and the OpenLAB CDS data processing system, detection was performed in positive and negative ion modes, respectively, under the following conditions:

Scan Mode	EMS	
Ionspray Voltage(IS)	5500V	-4500V
Curtain Gas(CUR)	40psi	30psi
GS1	80psi	80psi
GS2	70psi	70psi
TEM	375℃	375℃
DP	40V	-50V
EP	10V	-10V
CE	10V	-10V

The scan rate is 4000 Da/s; nitrogen is used in all gas lines; the mass scan range is 50–1500 (m/z); the scan time is 0.3 s; and the interval between scans is 0.02 s. Before performing instrumental analysis on the sample, the mass axis is first calibrated using a calibration solution.
